# Deciphering the Chemistry of Condensed Aromatic “Black” Carbon and Nitrogen in Amazonian Anthrosols

**DOI:** 10.1021/acs.est.5c09658

**Published:** 2025-08-05

**Authors:** João Vitor dos Santos, Aleksandar I. Goranov, Laís G. Fregolente, Marcia C. Bisinoti, Zhenhuan Sun, Klaus Schmidt-Rohr, Patrick G. Hatcher

**Affiliations:** † Department of Chemistry and Biochemistry, 6042 Old Dominion University, Norfolk, Virginia 23529, United States; ‡ Brazilian Nanotechnology National Laboratory, Brazilian Center for Research in Energy and Materials, Campinas 13083-970, Brazil; § Department of Chemistry and Environmental Sciences, 28108 São Paulo State University, São José do Rio Preto 15054-000, Brazil; ∥ Department of Chemistry, 427969 Brandeis University, Waltham, Massachusetts 02453, United States

**Keywords:** Amazonian anthrosols, condensed aromatic “black” carbon (ConAC), condensed aromatic “black” nitrogen (ConAN) oxidation, nitrogen incorporation

## Abstract

Amazonian anthrosols are renowned for their high fertility and dark color, properties primarily attributed to the abundance of condensed aromatic carbon (ConAC) in the soil organic matter. ConAC, commonly referred to as black carbon, play a key role in the stability and nutrient retention of these soils. However, the processes governing the formation of ConAC and its transformation into oxygenated derivatives remain poorly understood. In this study, we used multiple analytical platforms to investigate the chemistry of ConAC-rich humic acids (HA) extracted from *Terra Mulata de Indio*, a type of Amazonian anthrosol. The results reveal that ConAC are predominantly nonprotonated and consist of approximately 4–10 condensed rings. These structures exhibit varying degrees of oxygenation (1–24 oxygen atoms), suggesting that they are produced through oxidative processes. Approximately 20% of ConAC contain nitrogen atoms, referred to as ConAN (condensed aromatic nitrogen), which are part of either heterocyclic ring systems (commonly termed black nitrogen) or present as amine functional groups. As a result, we conclude that HA in Amazonian anthrosols contain polycyclic N-containing aromatic acids (PolyNARA), likely formed through combined charring of plant and animal biomass, abiotic nitrogen incorporation, and/or other soil processes. The mechanisms governing the formation, persistence, and transformation of PolyNARA in Amazonian anthrosols warrant further investigation, particularly given their potential implications for global carbon and nitrogen biogeochemical cycling.

## Graphical abstract

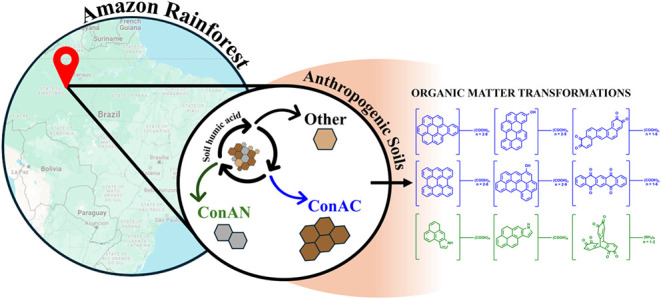

## 1 Introduction

The Amazon rainforest is characterized by its high humidity, consistent warmth, and heavy rainfall throughout the year.^1^ Many Amazonian soils are Ferrasols and Acrisols, which are inherently acidic, clay-rich, carbon- and nutrient-poor soils,^2^ properties largely attributed to the rapid leaching and erosion caused by heavy rainfall. Historically, the addition of large amounts of charred plant and animal biomass transformed these nutrient-poor soils into what is now referred to as black anthrosols or “*Terra Preta de Indio*” (TPI).^3^ The organic matter in these soils is highly aromatic, exhibits double the amount of carbon relative to Ferrasols and Acrisols, has high fertility, and, by definition, must contain ceramic and other archeological artifacts.^4–6^

Co-located with the TPI soils is another group of soils termed *Terra Mulata de Indio* (TMI), which are characterized by a dark brown color, lacking the black hue and artifacts characteristic of TPI, yet containing similar levels of carbon.^7,8^ There has been a growing fascination within the scientific community with TPI and TMI soils driven not only by archeological inquiries into the historical inhabitants of the Amazon region, but also by the prospect of deciphering and replicating the methods through which pre-Columbian societies permanently converted Ferrasols and Acrisols (soils of low agricultural productivity) into TPI and TMI soils of high fertility and productivity.^4,7,8^

The high aromaticity of organic matter in TMI and TPI soils has been attributed to the incorporation of charcoal, often referred to as black carbon.^4,9^ Structurally, charcoal is primarily comprised of condensed aromatic carbon (ConAC).^10^ Studies on Amazonian anthrosols have shown the presence of polycyclic aromatic acids suggesting that ConAC structures become oxygenated over time in the soils. Peripheral aromatic units of large ConAC molecules can oxidize to yield phenolic, carbonyl, and quinone units leading to eventual conversion to carboxylic groups,^11–14^ forming a polycondensed aromatic backbone that is heavily carboxylated.^4^ Oxidation starts at the surface of charcoal particles and propagates to the core over time.^15^ The formation of carboxyl groups makes the resulting oxygenated ConAC compounds more hydrophilic and introduces chemical reactivity that can have implications for nutrient cycling.^16^ An alternative, though less probable, explanation for the observed carboxylated ConAC structures is substantial amounts of oxidized organic matter becoming absorbed onto ConAC sheets.^15^

It is not only the carbon, but also the nitrogen cycle in soils that is of paramount importance for maintaining ecosystem health and productivity.^17^ There is growing research on how nitrogen transforms following environmental disturbances such as wildfires. One current perspective is that during wildfires nitrogen in compounds containing amide groups (i.e., CONH_2_, CONH-R, or CONR_2_ moieties) undergoes transformation into heterocyclic nitrogen (e.g., in the forms of pyrrole, pyridine, indoles). Such compounds can be classified as condensed aromatic nitrogen (ConAN) and are generally referred to as “black nitrogen” throughout the literature.^18^

However, contrary to the widespread belief that amides in the environment are mainly of biological origin and that ConAC and ConAN compounds form primarily at high temperatures during fires, studies have shown that these compounds can form abiotically, even in the absence of fire.^19–22^ Despite their potential importance, these abiotic processes in soils remain underexplored. This study does not aim to resolve which pathway, pyrogenic or nonpyrogenic, contributes more to the formation of ConAC and ConAN. Rather, we acknowledge that both hypothesized pathways, solely or simultaneously, could play a role in the formation of these compounds in soils.

While Amazonian anthrosols have been the subject of intensive research for decades, the detailed chemistry behind the formation and transformations of ConAC in these soils remains poorly understood. Much of the existing research has focused on the carbon sequestration potential of ConAC and its role in nutrient cycling and cation exchange capacity, often examining these compounds in broad terms without addressing the specific molecular mechanisms involved in their formation and transformations.^4,5,23,24^ Although significant progress has been made in characterizing ConAC’s structural properties through techniques like solid-state nuclear magnetic resonance (NMR),^4,5,9,25^ our study builds upon this foundational knowledge by using a method for characterization of individual molecular species, namely electrospray ionization-Fourier transform-ion cyclotron resonance-mass spectrometry (ESI-FT-ICR-MS). To our knowledge, this is the first study to employ ESI-FT-ICR-MS to investigate the molecular make-up and transformation pathways of both ConAC and ConAN in Amazonian anthrosols. This approach allows one to decipher the potential biogeochemical relationships between individual molecules and can also identify and assess species containing non-oxygen heteroelements such as nitrogen. We supplement the molecular fingerprinting from ESI-FT-ICR-MS with solid-state NMR, Raman, and X-ray photoelectron spectroscopies to provide comprehensive structural information, and perform benzenepolycarboxylic acid and chemothermal oxidation analyzes to quantify the ConAC and ConAN fractions.

This study enhances our understanding of the formation and transformation of ConAC and ConAN in TMI soils, providing valuable insights into the complex biogeochemical processes that govern soil fertility and resilience in tropical ecosystems. The findings could also guide soil management strategies aimed at boosting agricultural productivity by harnessing not only the well-known properties of ConAC but also those of ConAN, thereby improving soil structure, nutrient availability, and long-term soil health.

## 2 Materials and Methods

### 2.1 Soil Sampling

Amazonian anthrosols such as TMI are heavily protected due to their historical and ecological significance. Acquiring such soil samples is subject to strict legislation, making sampling across multiple sites quite challenging. Despite these limitations, samples from three distinct soils in the Amazon basin were obtained (Itacoatiara City, Amazonas State, Brazil). The first soil (TMI-1) was sampled from an area with native vegetation, sparse trees and plants (3 °04′05.4″ S and 58 °33′51.11″ W). The second sample (TMI-2) was taken from an undisturbed area, with native and dense vegetation cover (3 °04′05.17″ S and 58 °34′11.68″ W). The third sample (TMI-3) was collected in proximity to agricultural lands (3 °03′59.15″ S and 58 °27′04.64″ W). The varied land cover and anthropogenic influences on these soils make this data set sufficiently robust to explore the biogeochemistry of TMI soils.

Soil samples were collected from the surface horizon (0 to 30 cm). They were air-dried, ground, and sieved through a 2 mm mesh for removal of plant debris. The soil’s pH, moisture, ash, organic matter, and elemental contents (Table S1) were determined using standard methods (see Section 1 of the Supporting Information (SI)).

### 2.2 Humic Acid Extraction

Humic acid fractions (HA) were isolated from soils following the protocols outlined by the International Humic Substances Society.^26^ Briefly, 100 g of soils were extracted with 1 L of a 0.1 M NaOH (at soil-to-extractant ratio of 1:10) under nitrogen flow for 4 h. The extracted solutions were acidified using 6 M HCl until pH reached ∼2 resulting in precipitation of the HA fraction while the fulvic acid fraction remained in the supernatants. The separated HA were obtained through centrifugation. To eliminate inorganic constituents, the HA were treated with a solution of 0.1 M HCl and 0.3 M HF, which was followed by dialysis and subsequent freeze-drying.

### 2.3 Analytical Characterization

Carbon (C%), nitrogen (N%), and hydrogen (H%) contents in the three HA were determined using an elemental analyzer and published previously.^7^ Bulk structural characterization of carbon and nitrogen functional groups was done using solid-state ^13^C and ^15^N nuclear magnetic resonance spectroscopy (NMR; see Sections 2.1 and 2.2 of the SI), X-ray photoelectron spectroscopy (XPS; see Section 2.3 of the SI), and Raman spectroscopy (see Section 2.4 of the SI). Molecular-level characterization was done using electrospray ionization–Fourier transform–ion cyclotron resonance–mass spectrometry (ESI-FT-ICR-MS; see Section 3 of the SI).

The ConAC fraction was also quantified using two different approaches. The first one is the benzenepolycarboxylic acid (BPCA) molecular markers method, which uses a nitric acid thermochemolysis to convert ConAC to benzenehexacarboxylic acid (B6CA) and benzenepentacarboxylic acid (B5CA) markers, which are used for back-calculating the ConAC present in the sample (see Section 4.1 of the SI). The second method for quantifying ConAC is the chemothermal oxidation (CTO) method, which quantifies the thermally refractory fraction of organic matter in the sample (see Section 4.2 of the SI). Because this method depends on elemental analysis, it quantifies condensed aromatic nitrogen (ConAN) as well.

All analytical methods were performed following standard operating procedures unless stated otherwise, with relative standard deviations of replicate measurements of samples and/or of controls within standard reproducibility limits.

## 3 Results and Discussion

### 3.1 Condensed Aromatic “Black” Carbon (ConAC) in Humic Acids from Amazonian Anthrosols

TMI-1 and TMI-3 HA are C-rich (with C% of 51.24 and 50.22% respectively, Table S2) and H-deficient (with H/C ratios of 0.89 and 0.88, respectively, Table S2). This is typical for soil extracts from dark Amazonian soils.^27^ The H/C ratios <1.00 suggest high aromaticity. By contrast, TMI-2 contains less carbon (39.62%, Table S2) and is less aromatic (H/C = 1.23). This difference is likely due to a greater input of fresh, noncarbonized biomass at the TMI-2 site, which dilutes the contribution of ConAC and brings the H/C ratio closer to that of typical tropical HA.

Solid-state ^13^C NMR spectroscopy using direct polarization (DP) was employed to quantitatively assess the bulk structure and chemical composition of the HA (Figure 1; black lines). The resulting spectra were consistent with those reported in prior studies of TMI and TPI soils.^4,5,7,25^ Despite purification, residual ash remained in the HA (Table S2). Higher ash content, as in TMI-2 and −1, led to weaker NMR signals. After correcting for spinning sidebands, TMI-1 and TMI-3 showed nearly identical chemical compositions (Table S3). The ^13^C spectra are dominated by aromatic (aryl-C) and carboxyl (COO) signals (Figure 1). Aryl-C is the dominant structural constituent, with TMI-1 and TMI-3 containing 54.1 and 54.8% aryl-C, respectively, while TMI-2 contains 38.5% (Table S3). These results support previous H/C-based interpretations and confirm the high aromaticity of these materials. Furthermore, all three HA contain large contributions of carboxyl/amide (COO/N–C=O) groups (15.9, 20.2, and 15.5% for TMI-1, TMI-2, and TMI-3, respectively). The strong carboxyl signal, combined with low aliphatic-C content (10–105 ppm), suggests that aryl and carboxyl groups are structurally associated, likely as polycarboxylated condensed aromatic carbon structures, as previously observed in TPI soils.^4,5^

**Figure 1:**
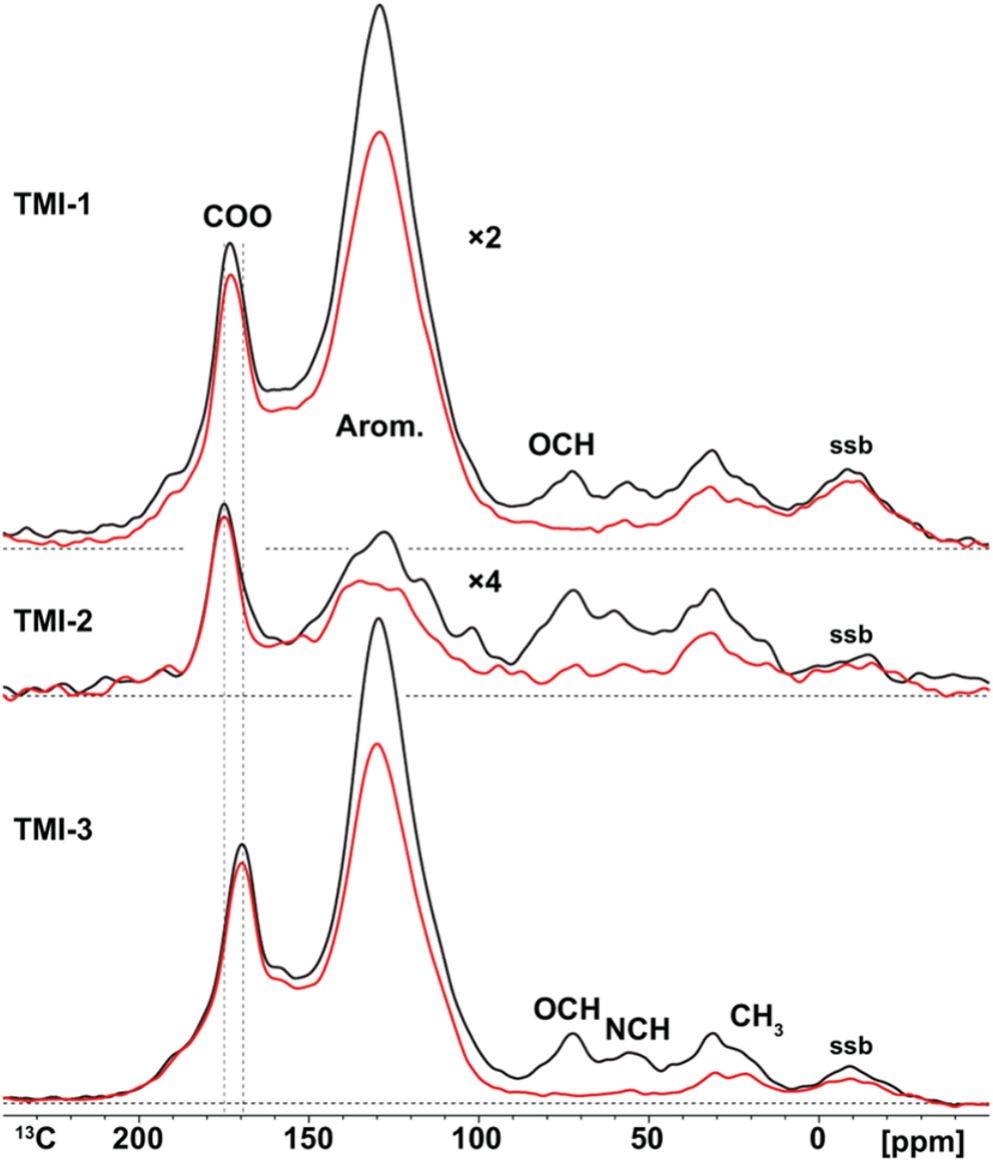
Quantitative, direct polarization ^13^C NMR spectra at 14 kHz of TMI humic acids (black lines) and spectra acquired with dipolar dephasing showing nonprotonated and mobile functional groups (red lines). The spectrum of TMI-1 was scaled 2× and TMI-2 4× for better visualization.

Quantitative spectral deconvolution further resolved functional group contributions, estimating relative proportions of charcoal-, lignin-, lipid-, protein-, and carbohydrate-like material.^28^ Charcoal-like material dominated all samples—79.4, 53.0, and 80.2% for TMI-1, TMI-2, and TMI-3, respectively, reinforcing that aryl-C arises primarily from ConAC structures (Table S4). The presence of ConAC can explain the high recalcitrance of soil organic matter in TPI soils.^25^ The next most abundant component was protein-like material, likely from fresh plant inputs, especially in TMI-2 (4.0, 16.5, and 3.8% for TMI-1, TMI-2, and TMI-3, respectively) (Table S4).

Additional NMR analysis using dipolar dephasing allows for selectively characterizing nonprotonated carbon functionalities (COO/N–C=O, C–C) as well as highly mobile aliphatic moieties such as methoxy groups (e.g., OCH_3_ in lignin). The near-complete suppression of signals at 72 and 103 ppm indicates that O-alkyl-C groups are protonated and likely derived from non-mobile carbohydrate structures (Figure 1; red lines). Spectral integration revealed that 79, 78, and 80% of aryl-C in TMI-1, TMI-2, and TMI-3, respectively, are not protonated (Table S5). This high proportion of nonprotonated aryl-C, centered near 130 ppm, is characteristic of fused aromatic ring systems.^5^

Aromatic cluster size was evaluated using two complementary solid-state ^13^C NMR approaches that provide distinct yet synergistic insights into the molecular architecture of ConAC in TMI HA. The first method relies on spectral analysis to estimate the fraction of edge-positioned aromatic carbons (χ_edge_), which reflects how many aromatic carbons are located internally of a condensed cluster versus on its periphery.^29,30^ Smaller aromatic clusters have a higher proportion of edge carbons, while more condensed clusters have more internal positions. From this, a lower bound on cluster size (*n*_C,min_) can be calculated, representing the minimum number of aromatic carbons needed to achieve the observed χ_edge_. Detailed calculations are provided in SI Section 2.1. The derived *n*_C,min_ values were: 10 for TMI-1 (indicative of at least two fused rings), 5 for TMI-2 (consistent with a monoaromatic structure), and 11 for TMI-3 (suggesting at least three fused rings) (Table S5).

The second technique, long-range ^1^H–^13^C dipolar dephasing, offers a dynamic and more holistic view of aromatic condensation. This method measures the rate at which nonprotonated aromatic carbon signals decay due to dipolar interactions with nearby protons. Slower dephasing rates imply longer average ^1^H–^13^C distances, indicating fewer protonated carbons and more condensed aromatic domains.^29^ This method estimates the average cluster size, which is usually larger than the minimum from χ_edge_. Figure 2a–c shows the dephasing series for all samples: TMI-3 yielded high-quality curves; TMI-1 showed moderate quality, likely due to interference from unpaired electrons; and TMI-2 exhibited poor signal-to-noise even after extended acquisition. Figure 2d compares normalized dephasing curves of the TMI samples with reference materials, including lignin, Terra Preta humic acids, pyrolysis char, and gasification char.^5,29,30^ As expected, lignin, which is dominated by monoaromatic units, underwent rapid signal loss, whereas the TMI samples, particularly TMI-3, retained significant signal intensity over time. At 0.5 ms, about 50% of the TMI HA signals remained; at 1.5 ms, ∼20% persisted; and at 2.5 ms, TMI-3 still retained ∼5% of its signal, consistent with the presence of large, highly fused aromatic clusters.

**Figure 2:**
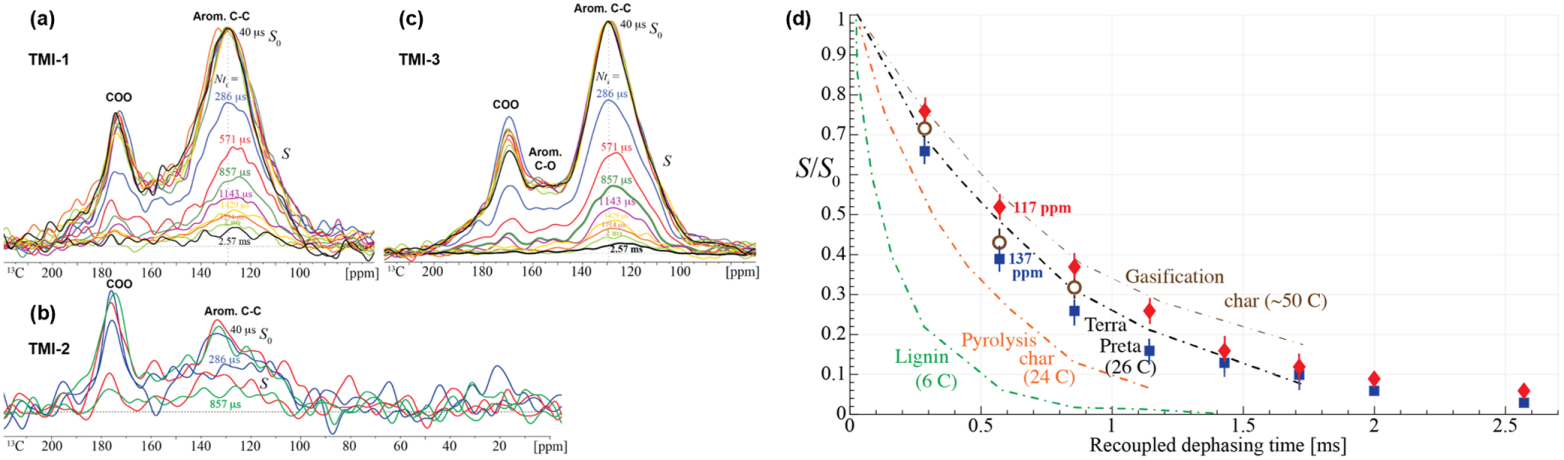
Recoupled long-range ^13^C­{^1^H} dephasing analysis of humic acids. Series of ^13^C NMR spectra of nonprotonated carbons in (A) TMI-1, (B) TMI-2, and (C) TMI-3 were acquired at 7 kHz MAS. Moderate *T*_2C_ relaxation of the aromatic carbons was corrected for by scaling each *S* and *S*_0_ pair such that the *S*_0_ spectrum was matched to the initial aromatic intensity. At long dephasing times, the peak maximum is seen to be shifted by about −5 ppm. The right panel (D) shows the normalized intensity of nonprotonated aromatic carbons in TMI-2 (open circles) and TMI-3 (blue filled squares measured at 137 ppm and red filled diamonds at 117 ppm) after the indicated duration of recoupled ^13^C­{^1^H} dipolar dephasing. Dashed-dotted lines, from left to right, indicate dephasing rates reported in the literature for lignin, pyrolysis char, Terra Preta humic acids, and gasification char (from refs 5,29,30 ).

Notably, the dephasing curves of the TMI HA fell between the dephasing curves of the pyrolysis char and gasification chars (Figure 2d), which indicates that TMI ConAC contained somewhere between 24 and 50 carbon atoms, which would correspond to between 6 and 13 rings (Figure S1). Most notably, the average dephasing behavior aligns closely with that of Terra Preta soil HA, which has been modeled to contain clusters with 22 aromatic and 4 C=O carbons, equivalent to roughly 6 fused rings.^5^ Furthermore, TMI-3 showed differential dephasing between regions of the spectrum: the signal at 117 ppm decayed 1.35 times more slowly than at 137 ppm, implying heterogeneity in cluster size, with some domains likely reaching 30 aromatic plus C=O carbons, or approximately 8 fused rings. The COO peak (Figure 1) varied among the three HAs, shifting upfield in more condensed domains (e.g., TMI-3) and downfield in more oxidized environments (e.g., TMI-2), further reflecting aromatic cluster heterogeneity.

Together, these two methods offer a robust picture of aromatic cluster architecture. The χ_edge_-based minimum estimates constrain the smallest plausible cluster size, while the long-range dipolar dephasing analysis reveals the broader distribution and average extent of condensation. Their combined use confirms that while smaller units exist, the dominant structures in TMI-1 and TMI-3 are large, fused aromatic domains comparable in size to those in neat black carbon-like materials.

Based on all spectroscopic and molecular results TMI-2 distinguishes itself from TMI-1 and TMI-3 with higher O-Alkyl-C and Alkyl-C contributions (18.2 and 18.9%, respectively; Table S3). Upon deconvolution (Table S4), TMI-2 has half the amount of charcoal-like material (26.3% relative to 54.8–63.5%) and substantial contributions from protein-like (22.6%) and carbohydrate-like material (17.3%). This is likely due to greater contributions of biopolymers from fresh plant litter as TMI-2 is sampled from an area with a dense and diverse native vegetation. This is further supported by the significant lignin-like contributions (13.8%), which is of negligible abundance in TMI-1 (4.9%) and is not present in TMI-3 (Table S4).

Additional structural information was obtained by XPS, which provides the elemental composition and chemical states of elements. Strong C, O, and N peaks (C 1s, O 1s, and N 1s, respectively) are observed for all three HA (Figure 3a for TMI-1; Figure S3a for all HA). High-resolution C 1s spectra (Figure 3b for TMI-1; Figure S3b for all HA) reveal the contributions of C=O, C–N, C–O, C–C/C–H, and C=C functional groups, as well as the π → π* shake-up satellite associated with aromatic structures in the HA. The C–C/C–H, which likely originate from aromatic compounds as suggested by the strong aryl-C resonances observed in NMR (Figure 1), are most abundant in TMI-1 and TMI-3. In contrast, C–O, likely originating from carbohydrates, are most abundant in TMI-2. High-resolution O 1s spectra (Figure 3c for TMI-1; Figure S3c for all HA) indicate contributions from C=O, C–OH, and C–O–C functional groups in HA structures. C=O and C–OH, likely from polycyclic aromatic acids, are predominant (>85%), while C–O–C (10–15%) likely arise from carbohydrates.

**Figure 3:**
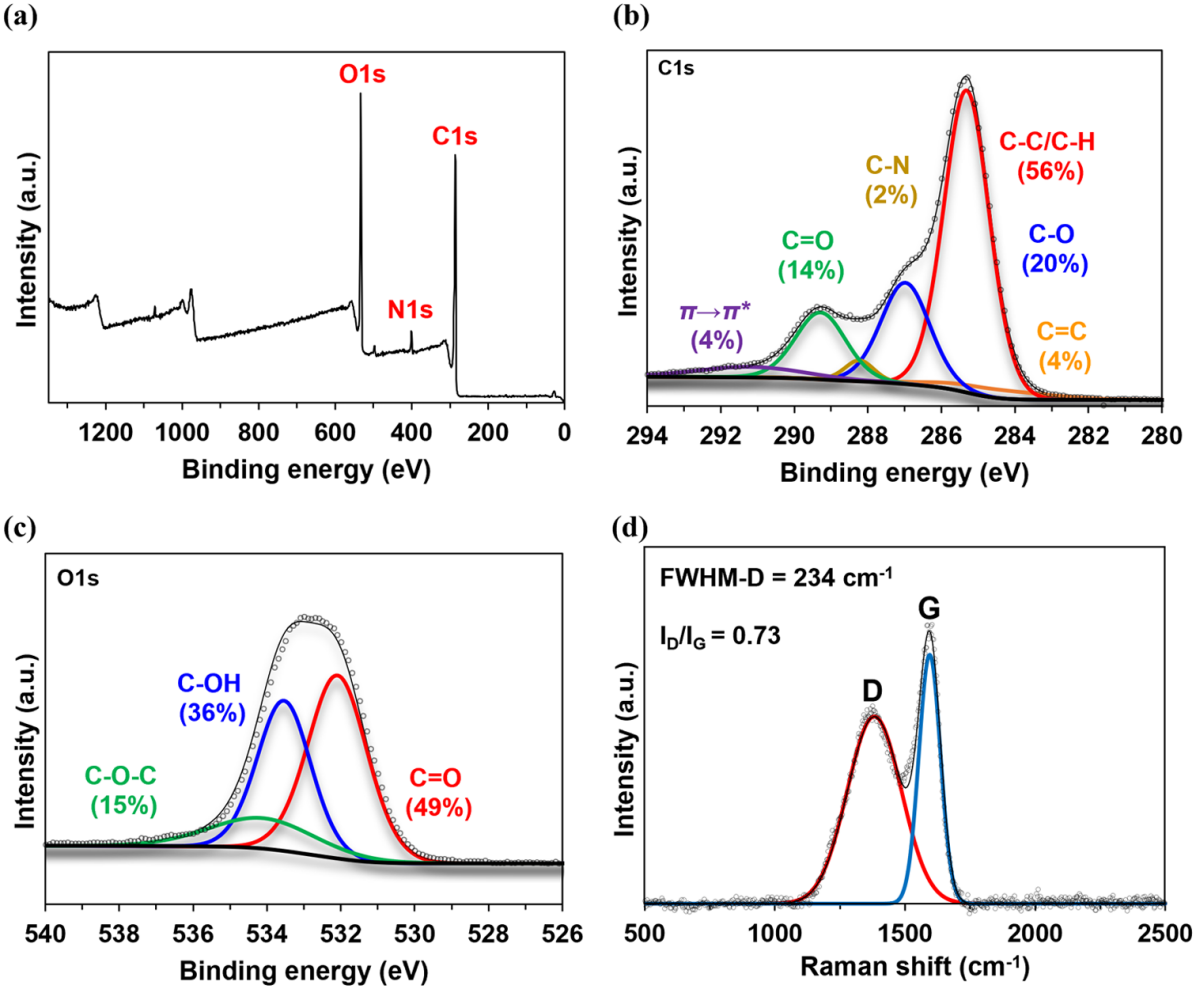
X-ray photoelectron spectroscopy (XPS) of TMI-1 humic acid. Survey wide-scan spectrum (a) shows the detected elements (annotated in red), whereas high resolution carbon (C 1s) and oxygen (O 1s) spectra are shown in (b) and (c), respectively. The Raman spectrum of TMI-1 humic acid is shown in (d) along with the calculated D-to-G ratio (*I*_D_/*I*_G_) and full width at half-maximum of the D peak (FWHM-D).

Further structural assessment was made by Raman spectroscopy. The HA exhibit D (∼1350 cm^–1^) and G (∼1580 cm^–1^) band profiles in their spectra (Figure 3d for TMI-1; Figure S4 for all HA). The G band is related to sp^2^ C=C stretching whereas the D band indicates structural disorder and the formation of defects in carbon network. Higher values of the D-to-G ratio for TMI-1 and TMI-3 (*I*_D_/*I*_G_ = 0.73 and 0.70) and broader peak full width at half-maximum of the D peak for TMI-1 and TMI-3 (FWHM-D = 234 and 224 cm^–1^) indicates more disordered carbon and a greater number of defects (e.g., heteroatoms) present in the carbon network structures, which is likely due to the high degree of oxidation these structures have experienced. The lower D-to-G and narrower D peak width in TMI-2 (*I*_D_/*I*_G_ = 0.56, FWHN-D = 124 cm^–1^) suggest fewer defects.

The composition of the three HA was further investigated by ESI-FT-ICR-MS providing molecular fingerprint maps, visualized using van Krevelen diagrams based on the H/C and O/C ratios of the detected molecular species (Figure 4).^31^ The three HA exhibit similar fingerprints containing primarily CHO-type molecules (77–90%) followed by N-containing compounds (7–18%). S- and P-containing compounds accounted for less than 5 and 1%, respectively (Figure S5a). The samples predominantly contain ConAC-type molecules (plotting below the modified aromaticity index (AI_MOD_) line of 0.67 on Figure 4) ranging from 62 to 87% of all detected species (Figure S5b). Interestingly, the O/C ratios of ConAC species vary from 0.1 to 0.7, suggesting the existence of different types of ConAC structures with varying degrees of oxidation with carboxyl or other O-containing moieties. Up to 20% of these ConAC compounds are N-containing (9.6–19.2%; Table S6) with practically negligible contributions from S- and P-containing ConAC (<0.2%; Table S6). Lignin-like compounds constitute 6–28% and carboxyl-containing aliphatic molecules (CCAM) account for 6–8% of the detected molecular species (Figure S5b). CCAM are species characterized with 0.85 ≤ H/C ≤ 2 and O/C ≤ 0.4, which includes various lipids (fatty acids, alicyclic compounds like sterols and hopanes, etc.) and other species often found in soils that are believed to be the end-products of lignin oxidation during humification.^32,33^ Formulas that do not fall into the ConAC, lignin, or CCAM categories could be categorized as sugar-, tannin-, and sulfonic acid-like compounds, but they collectively comprise 2–5% when combined and are thus, of negligible contributions (Figure S5b).

**Figure 4:**
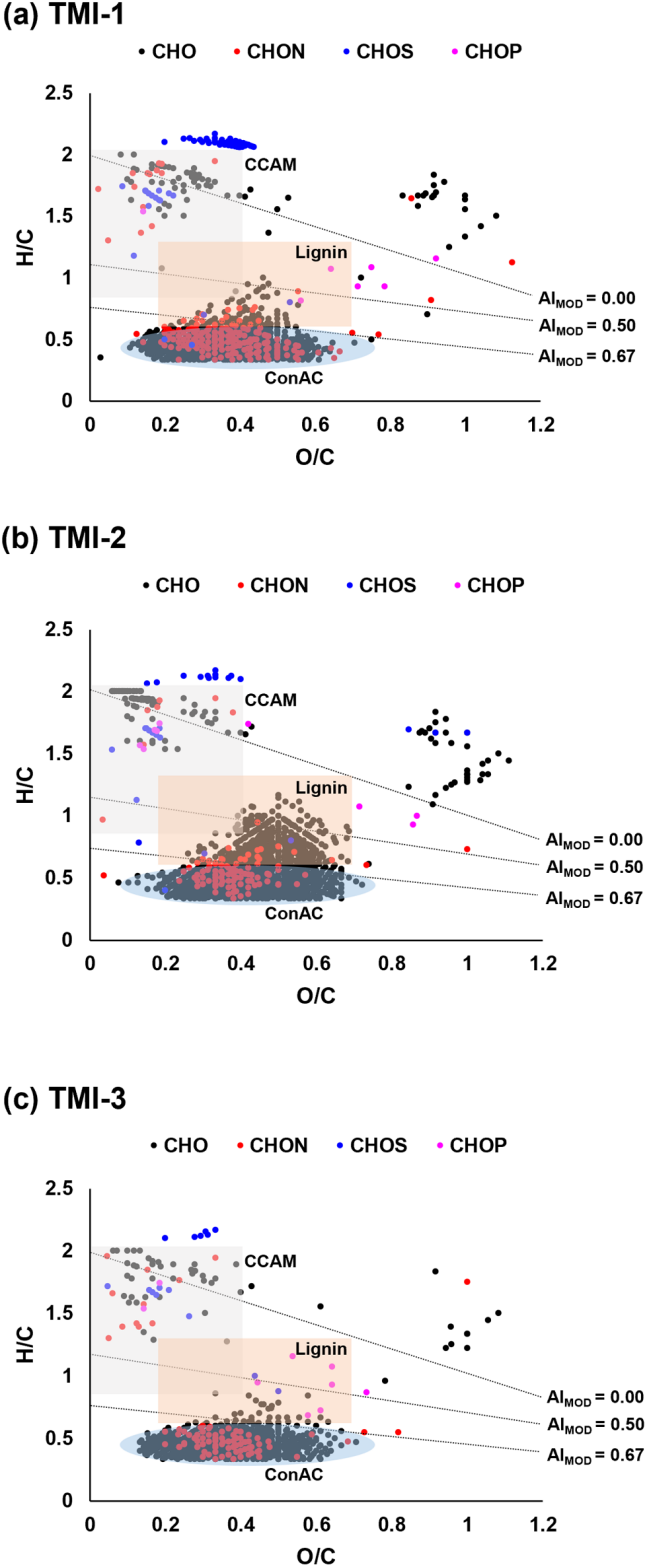
Van Krevelen diagrams of TMI humic acids: (a) TMI-1, (b) TMI-2, and (c) TMI-3. Dot color indicates the type of molecular formula (CHO in **black**, CHON in **red**, CHOS in **blue**, and CHOP in **pink**). Black lines separate the van Krevelen diagrams based on the modified aromaticity index (AI_MOD_), which is a proxy for the density of double-bonds in a molecule.

Given the substantial evidence supporting the presence of ConAC structures in these samples, obtaining quantitative confirmation is essential. To achieve this, we employed the benzenepolycarboxylic acid (BPCA) and chemothermal oxidation (CTO) methods, which are highly specific for quantifying ConAC. These two techniques have become the most widely used approaches for ConAC quantification in environmental matrices, providing reliable insights into its composition and abundance. Both of these methods have different analytical windows and capture ConAC of different degree of condensation.^34^ These quantitative results are compiled with semi-quantitative measurements from NMR and FT-ICR-MS that point toward ConAC presence (Table 1). The BPCA and CTO methods indicate that TMI-1 and TMI-3 contain large amounts of ConAC (up to 59.5%) while TMI-2 contains only 0.8–5.9%. These values are comparable to BPCA and CTO measurements of whole soils^35,36^ and HA fractions,^37^ including TPI soils.^38,39^ The differences in values must be due to the two different analytical windows for these methods–BPCA detects more charcoal-like ConAC (lower molecular size) whereas CTO detects more soot-like/graphitic ConAC (higher molecular size).^34,40^ The trends of quantified ConAC from BPCA and CTO methods generally agree with aromatic carbon measurements from NMR (Aryl-C + Aryl-O) or ConAC species from ESI-FT-ICR-MS. It must be noted that it is well expected that the absolute values do not match, because NMR and ESI-FT-ICR-MS are not able to quantify ConAC, but only give a measure associated with it. Collectively, these results lead to the conclusion that ConAC is a major contributor to HA of TMI soils.

**Table 1:** Condensed Aromatic Compounds (ConAC) Quantified by Benzenepolycarboxylic Acid (BPCA) andChemothermal Oxidation (CTO) Methods Contextualized with Relevant Measurements from Solid-State Nuclear Magnetic Resonance (NMR) and Electrospray Ionization–Fourier Transform–Ion Cyclotron Resonance–Mass Spectrometry (ESI-FT-ICR-MS)

						ConAC from ESI-FT-ICR-MS	
	ConAC from BPCA (%)	ConAC from CTO (%)	aryl C from NMR (%)	nonprotonated aryl C from NMR (%)	charcoal-like C from NMR (%)	formulas	% of all formulas
TMI-1	37.74 ± 0.04	12.6 ± 2.3	65.6	51.6	79.4	1139	82
TMI-2	5.99 ± 0.20	0.8 ± 0.3	45.8	35.9	53.0	957	62
TMI-3	30.93 ± 1.11	59.5 ± 5.5	65.8	52.8	80.2	862	87

### 3.2 Oxidative Transformation of ConAC in TMI Humic Acids

ConAC is characterized by rings of carbon atoms linked together in a condensed and stable form and are known to be resistant to biological decomposition and persist in soils over extensive periods of time.^41,42^ However, due to the existence of strong oxidation gradients in soils, it is expected that ConAC structures will undergo oxidative transformations over time^43^ that likely retain the majority of ConAC core and only alter the periphery of ConAC structures. ConAC oxidation has been proposed to be a continuum of different chemical stages:^13,44^ (1) hydroxylation (addition of OH groups to peripheral aromatic units of ConAC), (2) oxidation to quinones, carbonyls (aldehydes and ketones), and carboxylic acids, and (3) decarboxylation (removal of COO groups) to yield CO_2_ emissions. Oxidation may be (1) photochemical, driven by sunlight in surficial soil systems;^45^ (2) driven by dark Fenton reactions producing various reactive oxygen species (^•^OH radicals, superoxide, etc.) in soil horizons, particularly those rich in minerals,^46^ and (3) microbial, driven by extracellular enzymes like peroxidases and laccases that release reactive oxygen species.^47^ Thus, it is to be expected that ConAC species have experienced some oxidation in the TMI soils. The varying data from about these three HA suggest a diversity of compounds in these soils. This is further assessed by exploring the oxygen classes of ConAC molecular formulas (Figure S6). It is evident that ConAC compounds contain 1–24 oxygen atoms (total O*_*x*_* range: O_1_–O_24_), but the species with 7–9 oxygen atoms (O_7_–O_9_) are most abundant across all samples. The O/C ratio of ConAC molecular formulas varies from 0.1 to 0.7, and the carbon oxidation state for ConAC varies from −0.5 to +1.0 (Figure S7). Collectively, these results indicate that TMI HA contain a broad distribution of partially to highly oxidized ConAC structures.

To further explore ConAC oxygenation, Kendrick mass defect (KMD) analyses of ESI-FT-ICR-MS data were performed to further unveil the potential reaction pathways of ConAC oxidation during humification (Figure 5 for TMI-1; Figure S9 for all HA). KMD carboxyl (COO) series (Figure 5a) show that numerous formulas are aligned on the same horizontal lines, which indicates they are part of a homologous series. Compounds in such series only differ by the number of carboxyl groups. For example, the KMD series with a value of 0.1905 (upper series in Figure 5b) contain seven formulas (C_30_H_14_O_4,_ C_31_H_14_O_6,_ C_32_H_14_O_8_, C_33_H_14_O_10_, C_34_H_14_O_12_, C_35_H_14_O_14_, C_36_H_14_O_16_), all differing by one carbon and two oxygen atoms (i.e., COO). Out of all formulas, 80% are found to be a part of such a series (Figure 5a) and interestingly, the precursors (formulas which are first in the series, i.e., with lowest number of COO groups) can be found across the entire van Krevelen space (Figure 5c). This suggests that both addition and removal of COO groups exist, which is expected based on the previously proposed oxidation mechanism for ConAC.^13^ Both biotic and abiotic pathways may contribute to the formation and transformation of these polycarboxylated ConAC species. The KMD analysis results for TMI-2 and TMI-3 are similar to those for TMI-1 (Figure S9) suggesting that strong oxidation processes exist during the humification process in all three TMI soils.

**Figure 5:**
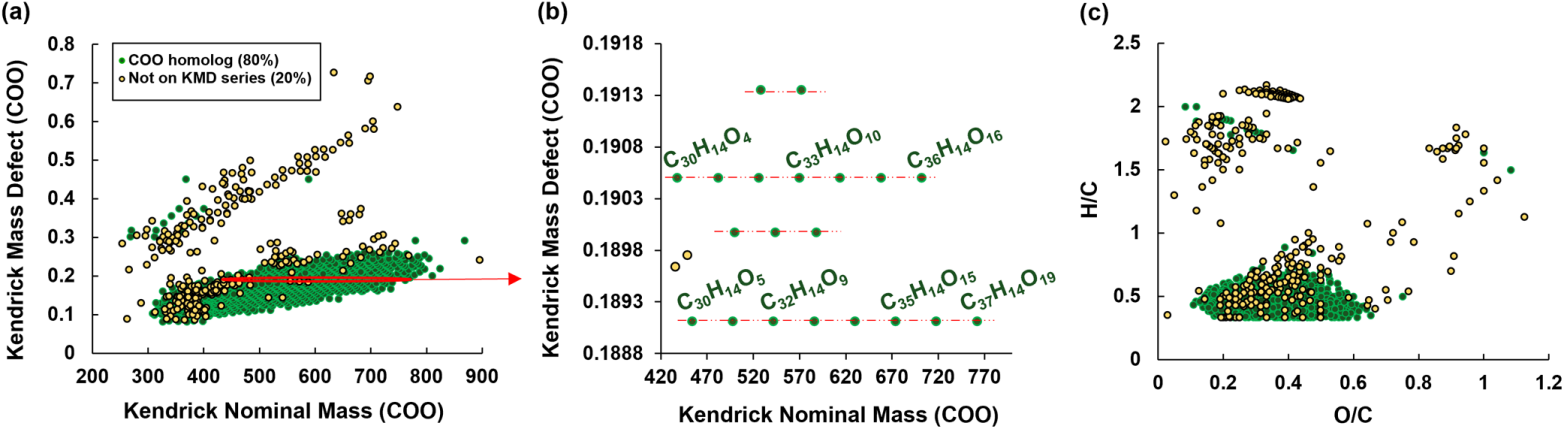
Kendrick mass defect (KMD) analysis using carboxyl (COO) series of TMI-1 HA formulas derived from ultrahigh resolution mass spectrometry. Formulas in green represent species potentially involved in oxygenation reactions. Formulas that are not part of KMD series are colored in yellow. Panel (a) shows the whole KMD plot while panel (b) shows an expanded region. For clarity, only the molecular formulas for KMD = 0.1891 and 0.1905 series are labeled. Panel (c) shows the van Krevelen distribution of the formulas.

Due to the complex nature of soil organic matter, achieving an accurate structural assignment to individual molecular species is impossible at present. However, by combining data from different techniques, one can speculate on potential molecular structures. Based on the structural and molecular characteristics observed in this study, we identify the ions with the highest intensity common to the three HA in our KMD analysis using COO series and design several potential structures of oxygenated ConAC compounds (Figure 6). Based on the KMD of 0.1905 (per KMD analysis; Figures 5 and S9), suggested structures contain a condensed aromatic core and 2–8 COOH groups (Figure 6a). The KMD of 0.1891 (per KMD analysis; Figures 5 and S9), additional oxygenated ConAC structures are proposed (Figure 6b). Besides highly condensed (circular catenation), aromatic cores with linear chain configuration are also suggested (Figure 6c). Notably, many of these likely structures contain quinone moieties (conjugated cyclic diones), which are key intermediates during ConAC oxidation. Quinones are highly reactive compounds due to their α, β-unsaturated carbonyl electrophilic centers. Quinones and hydroquinones have been identified as the most common redox-active couples in natural organic matter and pyrogenic carbon.^48,49^ The presence of quinones is suggested by NMR characteristics with a peak at 185–220 ppm (3.0–4.6%; Table S3).

**Figure 6:**
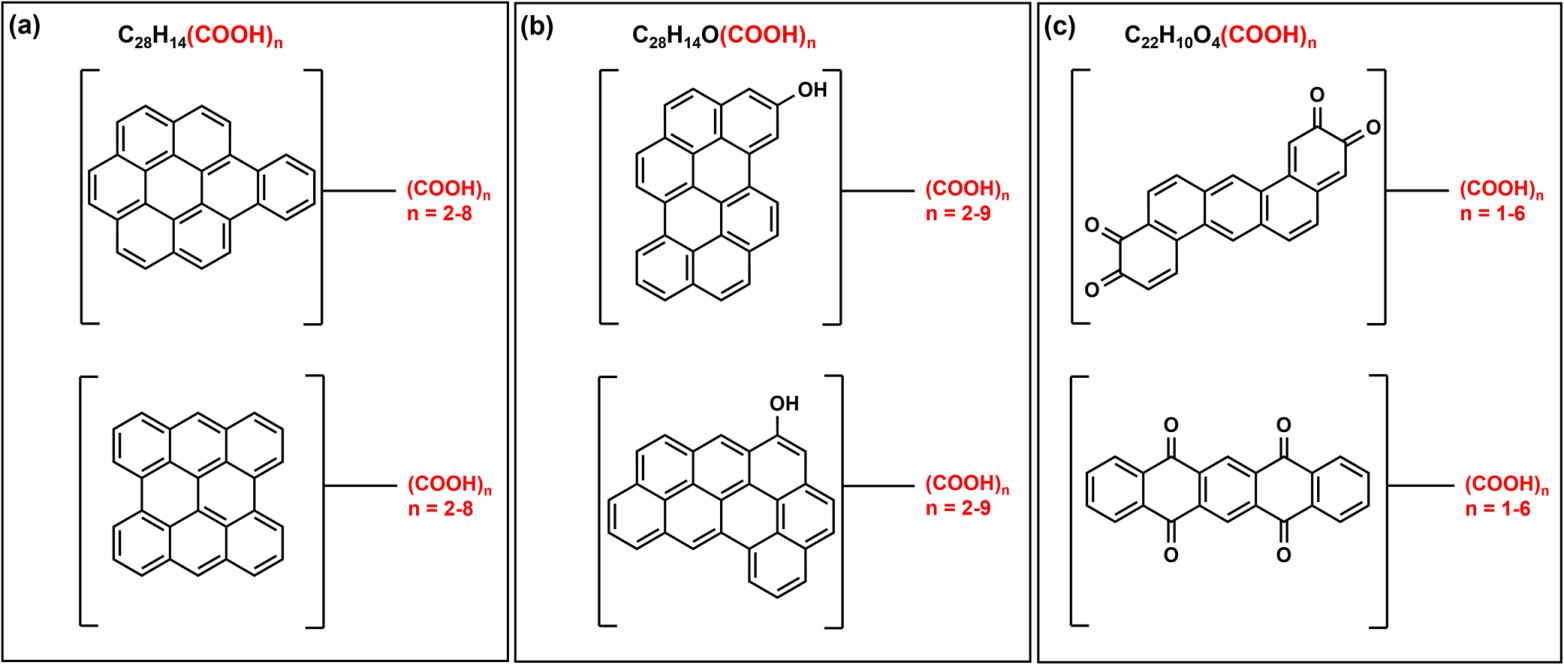
Possible structures for condensed aromatic carbon (ConAC) compounds found in TMI humic acids, which are also part of COO homologous series (Figure 5). Based on KMD analysis, the number of COO groups was determined and the type of condensed aromatic core that could yield a singly charged ion detected by ESI-FT-ICR-MS was inferred. Two structures per molecular formula are suggested. These example structures may exist in the samples as proposed or as other isomeric forms.

### 3.3 Nitrogen Immobilization into ConAC to Form “Black” Nitrogen

While the most important feature of ConAC structures in TMI appears to be their polycarboxylated peripheries, some of these structures are also found to contain nitrogen (Figure 4). ESI-FT-ICR-MS reveal that 9.6–19.2% of the detected ConAC species are N-containing (CHON-type formulas, Table S6). N-containing compounds are typically associated with proteinaceous molecules, which usually plot at H/C ≥ 1.5 and O/C < 0.55 in the van Krevelen space. The observed N-containing compounds in TMI HA plot at H/C < 1.0, and more specifically, below the AI_MOD_ = 0.67 threshold (Figure 4). This indicates that these CHON compounds are not proteinaceous and are of ConAC form.

Organic nitrogen in soils primarily exists in the form of amide- and amine-N.^50^ In regions susceptible to frequent wildfires, the heat transforms proteinaceous compounds into N-containing heterocycles, such as pyrrole-, pyridine-, and indole-type N, which can be collectively referred to as condensed aromatic nitrogen (ConAN).^51,52^ Some pyridine-N type structures can also be formed from pyrroles at higher pyrolysis temperatures via ring expansion.^53,54^ Often fire-derived nitrogen compounds are referred to as “black nitrogen”.^50,55^ It is generally recognized that elevated temperatures during burning are a prerequisite for the formation of ConAN.^55^

To characterize the type of nitrogen in TMI HA samples we applied solid-state ^15^N NMR, XPS, and quantified condensed aromatic nitrogen (ConAN) using the CTO method. The NMR spectrum has one peak centered at −263 ppm (Figure S2), which can be assigned as an amide-N.^56^ However, the observed amide-N peak does not exclude the presence of other nitrogen functionalities as the employed NMR technique (ramped-amplitude cross-polarization) is not quantitative and has a specific analytical window that poorly detects certain types of nitrogen such as heterocyclic nitrogen.^57^ High resolution N 1s spectra from XPS (Figure 7) allows for a better assessment of the contributions of different types of nitrogen.^58^ XPS reveals the presence of amide, pyrrole, amine, protonated amine, and pyridine moieties in the structures of TMI HA. TMI-1 shows a more diverse composition of nitrogen functionalities containing 14–25% of the five types of nitrogen moieties. By contrast, TMI-2 and TMI-3 have large contributions from pyrrole-like nitrogen (44 and 39%, respectively) followed by protonated amine (34 and 29%, respectively). Quantification of ConAN reveals that it can account for up to 61% of the nitrogen in the HA samples (Table S9). Ultrahigh resolution mass spectrometry reveals that N_1_ species are prevalent within ConAC of the three HA (Figure S8a), with N_1_O_7_ and N_1_O_8_ species being dominant (Figure S8b). Given that the O_7_–O_9_ classes are the most abundant within ConAC (Figure S6), this shows that ConAC units most commonly have one nitrogen immobilized in their structure.

**Figure 7:**
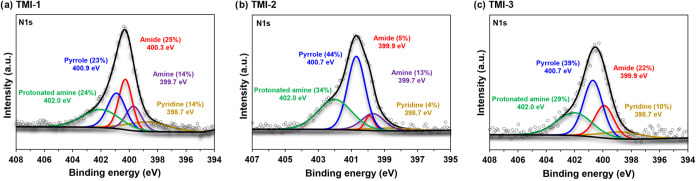
High resolution nitrogen (N 1s) spectra of TMI humic acid samples, where (a) is TMI-1, (b) is TMI-2, and (c) is TMI-3, obtained from X-ray photoelectron spectroscopy (XPS). Survey spectra are shown in the top panels (a) of Figure 3 and S3.

Many aspects of the biogeochemistry of ConAC in Amazonian anthrosols remain poorly understood, particularly the interactions between carbon and nitrogen cycles. In contrast to the existing literature, which characterizes oxidized ConAC simply as polycyclic aromatic acids,^4,5^ our research reveals that some of these compounds contain nitrogen moieties. We term these compounds polycarboxylic nitrogen-containing aromatic acids (PolyNARA). The prevailing belief is that ConAC in Amazonian anthrosols originates from pyrogenic activities carried out by pre-Columbian indigenous peoples, with the incorporation of charred animal biomass, such as bones, fish bones, and chelonian shells.^3,59^ These organic residues, which do contain nitrogen, along with charred plant biomass, may contribute to the formation of nitrogen-containing aromatic compounds (ConAN). After undergoing natural partial oxidation, these compounds can lead to the development of PolyNARA through both biotic^60^ or abiotic^61^ pathways.

To test if abiotic N immobilization could occur, we assessed KMD ammonia (NH_3_) series, which display molecular formulas that differ by one or more NH_3_ units (Figure 8 for TMI-1; Figure S10 for all HA). The data shows that 9% of the total formulas (40% of the CHON total formulas and 74% of the ConAC CHON formulas) could have been formed by the incorporation of NH_3_ into soil organic matter (Figure 8a). An example is shown at KMD = 0.4956 (Figure 8b) with C_20_H_8_O_6_ → C_20_H_11_O_6_N → C_20_H_14_O_6_N_2_. This scheme indicates that N immobilization of ammonia through Michael addition might be possible as shown previously.^62–68^ While ammonia was used to model a simplified system, protein-like compounds in the HA (Table S4) may also undergo immobilization through more complex environmental pathways.

**Figure 8:**
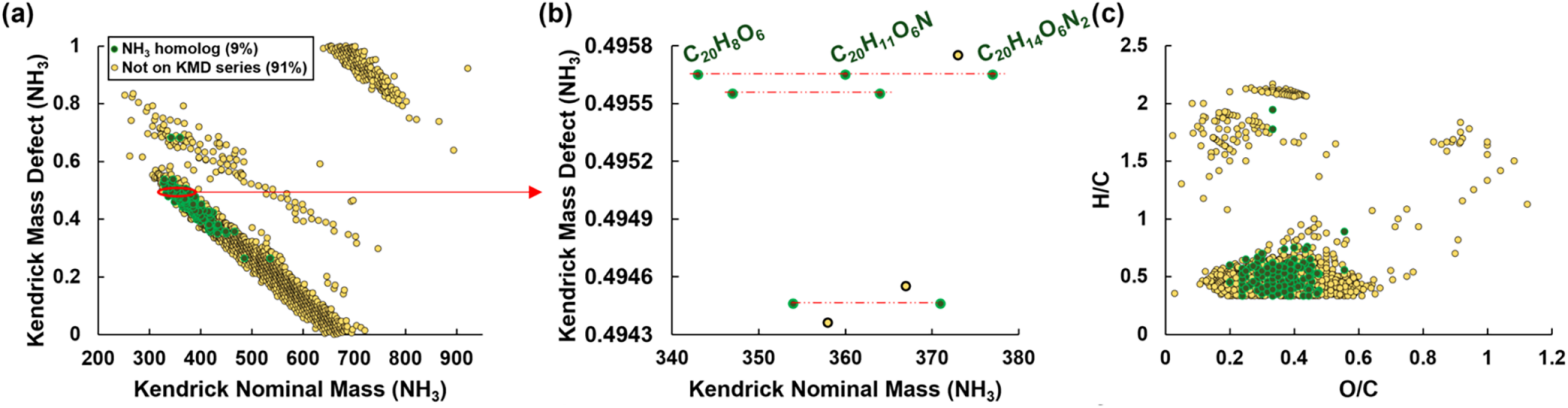
Kendrick mass defect (KMD) analysis using ammonia (NH_3_) of formulas of TMI-1 HA. Formulas in green represent species potentially involved in ammonia reactions. Formulas not part of KMD series are colored in yellow. Panel (a) show the whole KMD plots while panel (b) shows an expanded region. For clarity, only the molecular formulas for one of the series are labeled. Panel (c) shows the van Krevelen distribution of the formulas.

Based on the observed structural and molecular characteristics in this study, we have designed potential structures for PolyNARA (Figure 9). Utilizing ions exhibiting the highest intensity common to the three HA in our KMD analysis using the NH_3_ series, we formulated potential structures containing amino groups (Figure 9a). Additionally, PolyNARA likely features a polycyclic aromatic core with fused aromatic rings, as indicated by pyrrole signatures (per XPS data; Figure 7), and four COOH groups per ESI-FT-ICR-MS analysis, suggesting that N_1_O_8_ compounds are the most prevalent (Figure 9b,c). Triptycene quinones, such as the one depicted on top of Figure 9a, can also form via Diels–Alder cycloaddition between polycyclic aromatic hydrocarbons and benzoquinone, followed by an *in situ* oxidation facilitated by excess benzoquinone.^69,70^ A ConAC-like isomer like the bottom structure in Figure 9a may also exist. The pyrrole-containing PolyNARA proposed in Figure 9b,c may result from fire-related or nonpyrogenic radical processes.^20^

**Figure 9:**
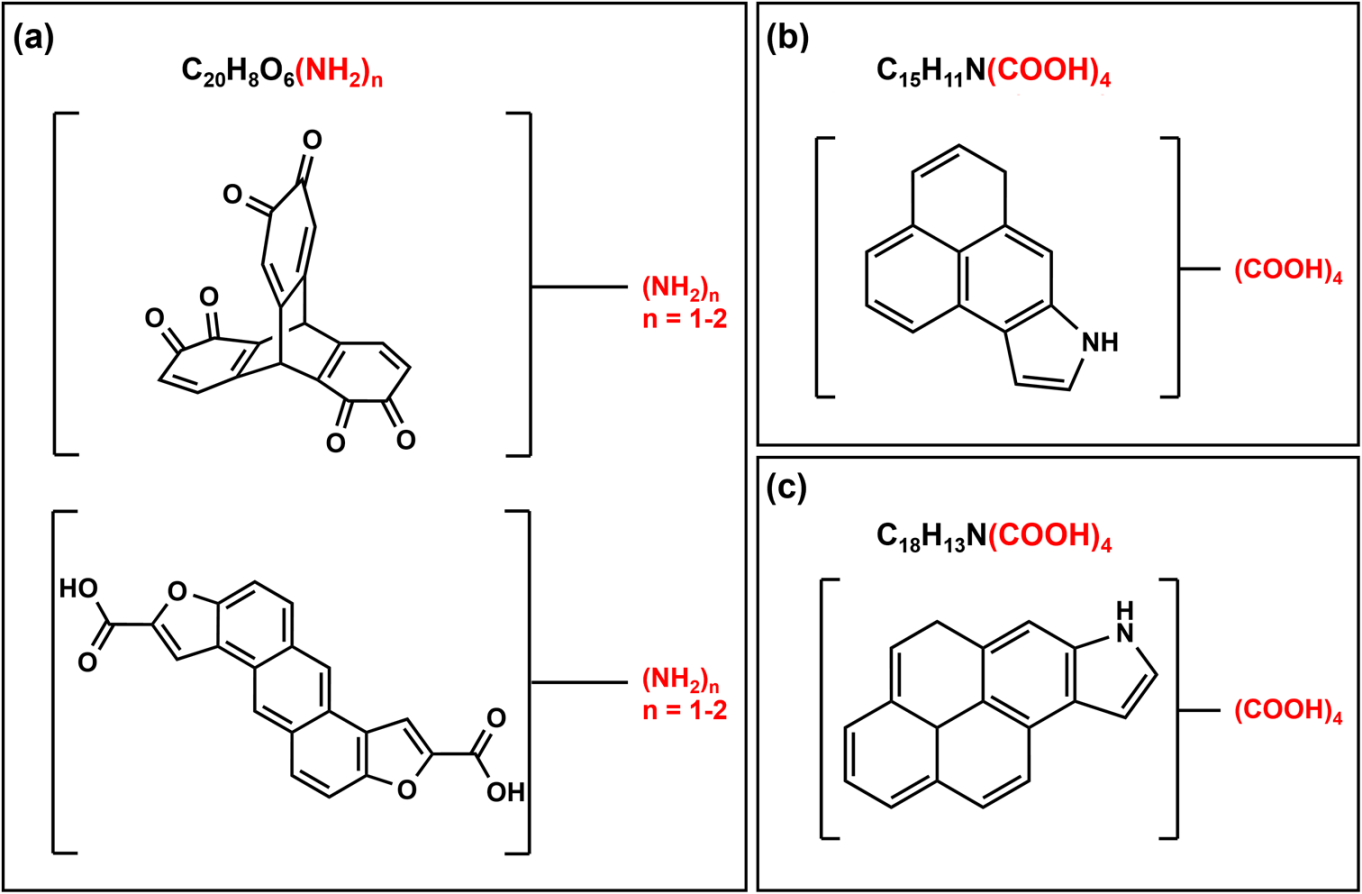
Potential structures for polycyclic N-containing aromatic acids (PolyNARA) identified in TMI humic acids. Panel (a) on the left shows two isomers suggested for the same molecular formula, which is part of the NH_3_ homologous series shown on Figure 8b. Panels (b, c) on the right propose structures based on the two most abundant ions with N_1_O_8_ type molecular formulas. These example structures may exist in the samples as proposed or as other isomeric forms.

### 3.4 Environmental Significance

Decades of research on Amazonian anthrosols have yet to provide a comprehensive understanding of the chemical dynamics surrounding condensed aromatic “black” carbon, ConAC. Previous insights remain limited, particularly concerning ConAC’s structural composition and its temporal evolution. Using ESI-FT-ICR-MS, we unveil its prevalence and structural diversity and explore the rarely studied condensed aromatic “black” nitrogen, ConAN, in Amazonian anthrosols.

It is known that ConAC in Amazonian anthrosols undergoes oxidation, which enhances cation exchange capacity and contributes to improved soil fertility. We provide a comprehensive overview of ConAC chemistry, proposing potential structures and discuss the reactions that may contribute to oxygenation observed worldwide in tropical systems. ConAC exhibits a spectrum of oxidation levels, ranging from relatively reduced to highly oxidized species. The diversity in oxidation states has significant implications for soil carbon stability, long-term storage, and fertility. Approximately 10% of ConAC appears less oxidized, potentially offering enhanced stability. Despite its varying degrees of oxidation, ConAC may actually remain a long-term carbon sequestration product, especially as some fraction of it is resistant to oxidation.^20^

Polycarboxylic N-containing aromatic acids (PolyNARA), the ConAN portion of oxygenated ConAC, are likely a crucial yet underexplored component in Amazonian anthrosols. These compounds, characterized by fused aromatic rings, pyrrole or amine functionalities, and carboxylic acid groups, likely play critical roles in soil fertility and the cycling of carbon and nitrogen. Due to their abundance of functional groups, PolyNARA can contribute to cation exchange capacity through electrostatic interactions involving deprotonated carboxyl groups. Additionally, their ability to chelate metal ions through specific coordination with oxygen- and nitrogen-based ligands may influence nutrient mobility and availability. PolyNARA formation may result from charred plant and animal biomass, as well as from abiotic nitrogen immobilization or other chemical reactions.^62–65^ The covalent bonds between ConAC and N-species may promote persistent nitrogen retention, making PolyNARA a long-term sink for carbon and nitrogen in soils. Overall, little is known about the structure of ConAN compounds, because this study marks their first exploration in Amazonian anthrosols. Future research should explore how organic matter retains nitrogen through covalent bonds, elucidate the underlying mechanisms, and assess the implications for global nitrogen biogeochemistry.

In addition, the trends observed in this study were consistent across all three HA samples, indicating that the examined biogeochemical processes may have broader applicability to similar anthrosols. Since HA molecules are generally resistant to seasonal variations,^71^ and the studied TMI soils encompass diverse land cover and anthropogenic influences, the findings presented here likely offer insights that are applicable across broader spatiotemporal scales.

## Supplementary Material



## Notes

Detailed descriptions of the experimental procedures and data analyses for soil and humic acid characterization; bulk physicochemical properties of soils and humic acids; structural characterization using solid-state ^13^C and ^15^N NMR, XPS, and Raman spectroscopy; molecular-level analysis using ultrahigh-resolution ESI-FT-ICR-MS; quantification of ConAC and ConAN via benzenepolycarboxylic acid and chemothermal oxidation methods; and comprehensive data tables on the composition and structure of ConAC (PDF)
