# Supplementary material for: Deciphering the Chemistry of Condensed Aromatic “Black” Carbon and Nitrogen in Amazonian Anthrosols
Source: Environ Sci Technol. 2025 Aug 5;59(32):17047–58. doi: 10.1021/acs.est.5c09658 (PMC12368991; doi:10.1021/acs.est.5c09658)
Supplement: Supplementary file 1 [file es5c09658_si_001.pdf]

Deciphering the chemistry of condensed aromatic "black" carbon and nitrogen in Amazonian anthrosols

João Vitor dos Santos[a*], Aleksandar I. Goranov[a], Laís G. Fregolente[b], Marcia C. Bisinoti[c], Zhenhuan Sun[d], Klaus Schmidt-Rohr[d], Patrick G. Hatcher[a*]

[a]Department of Chemistry and Biochemistry, Old Dominion University, Norfolk, Virginia 23529, United States
[b]Brazilian Nanotechnology National Laboratory, Brazilian Center for Research in Energy and Materials, Campinas, 13083-970, Brazil
[c]Department of Chemistry and Environmental Sciences, São Paulo State University, São José do Rio Preto, 15054-000, Brazil
[d]Department of Chemistry, Brandeis University, Waltham, Massachusetts 02453, United States

Corresponding authors (*): João Vitor dos Santos (j1dossan@odu.edu) and Patrick G. Hatcher (phatcher@odu.edu)

Summary: 23 pages, 10 figures, 11 tables.

Supporting Information (SI)

Section 1. Bulk characteristics of soils and humic acids 2
Section 2. Spectroscopic structural analyses 3
  Section 2.1. Solid-state ^{13}C nuclear magnetic resonance (NMR) 3
  Section 2.2. Solid-state ^{15}N NMR 6
  Section 2.3. X-ray photoelectron spectroscopy (XPS) 7
  Section 2.4. Raman spectroscopy 9
Section 3. Molecular fingerprinting using ultrahigh resolution mass spectrometry (ESI-FT-ICR-MS) 9
  Section 3.1. Instrumental Analysis 9
  Section 3.2. Data Analysis 10
  Section 3.3. Results 11
Section 4. Quantification of condensed aromatic carbon (ConAC) and nitrogen (ConAN) 17
  Section 4.1. Benzenepolycarboxylic acid (BPCA) analysis 17
  Section 4.2. Chemothermal oxidation (CTO) analysis 18
Section 5. Characteristics of ConAC and ConAN structures 20
References 21

Section 1. Bulk characteristics of soils and humic acids

Soil pH was measured by suspending the soil into ultrapure water in a proportion of 1:2.5 of soil:water[1] and measuring the pH directly using a benchtop pH-meter (MAPA 200, Marconi). Moisture content was measured by weight difference after drying samples in a drying oven at 110 °C for 16 hours. Ash content was determined by difference after burning samples in a muffle oven at a temperature of 750 °C for 4 hours. The organic matter (OM) content was determined by difference as shown in Eq. 1.[2]

$$\text{OM}\ (\%) = 100\% - \text{Moisture}\ (\%) - \text{Ash}\ (\%) \qquad \text{Eq. 1}$$

Elemental analysis on bulk soils and humic acids was performed using an elemental analyzer (2400 Series II CHNS/O, Perkin Elmer, Walther, Massachusetts, USA). Oxygen content was calculated by subtracting the C, H, N, S, and ash contents from the total weight. The C, H, N, S, and O contents were recalculated on an ash-free basis, and used to calculate the H/C, O/C and C/N atomic ratios. All analyses were done in triplicate.

Table S1. Soil pH, moisture, ash, organic matter, and elemental analysis weight fractions of TMI soils.

	pH	Moisture (%)	Ash (%)	Organic matter (%)	C (%)	H (%)	N (%)
**TMI-1**	5.70 ± 0.11	3.07 ± 0.24	87.72 ± 0.31	10.21 ± 0.54	4.91 ± 0.25	1.92 ± 0.10	1.21 ± 0.06
**TMI-2**	4.75 ± 0.06	0.83 ± 0.04	92.93 ± 0.14	7.24 ± 0.18	2.83 ± 0.14	1.52 ± 0.08	0.85 ± 0.04
**TMI-3**	6.05 ± 0.03	5.28 ± 0.19	84.64 ± 0.17	11.08 ± 0.11	6.46 ± 0.32	1.98 ± 0.10	1.24 ± 0.06

Table S2. Elemental composition of TMI humic acids.

	C (%)	H (%)	N (%)	O (%)	O/C	H/C	C/N	Ash (%)
**TMI-1**	51.24 ± 0.13	3.80 ± 0.11	3.41 ± 0.14	40.61 ± 0.62	0.59 ± 0.01	0.89 ± 0.02	17.80 ± 0.68	25.05 ± 0.53
**TMI-2**	39.62 ± 0.08	4.05 ± 0.19	4.04 ± 0.07	51.37 ± 0.39	0.97 ± 0.01	1.23 ± 0.06	11.38 ± 0.18	46.41 ± 0.67
**TMI-3**	50.22 ± 0.27	3.69 ± 0.01	2.97 ± 0.01	42.14 ± 0.08	0.63 ± 0.00	0.88 ± 0.00	19.95 ± 0.20	8.56 ± 0.57

Section 2. Spectroscopic structural analyses

Section 2.1. Solid-state ^{13}C nuclear magnetic resonance (NMR)

Solid-state ^{13}C NMR spectra were acquired using quantitative direct polarization (DP) on a Bruker Avance Neo 400WB NMR spectrometer. Samples were packed into 4 mm zirconia rotors with 3-mm high cylindrical glass spacers at the bottom and sealed with Kel-F caps. Spectra were recorded at a spinning rate of 14 kHz. Glycine was used for chemical-shift calibration, with the COO^- resonance set to 176.49 ppm. Spin-lattice relaxation times (T_{1H} and T_{1C}) were measured to determine the appropriate recycle delays. The T_{1H} relaxation times were extremely short (3, 17, and 8 ms for TMI-1, -2, and -3, respectively), indicating high concentrations of unpaired electrons (i.e., persistent radicals) in the samples. Accordingly, T_{1C} relaxation was also fast, and fully relaxed DP ^{13}C NMR spectra could be obtained with recycle delays of 2 s, 10 s, and 2 s for TMI-1, -2, and -3, respectively. In TMI-1 and -2, unusually large spinning sidebands were observed in ^{1}H and ^{13}C NMR spectra, which can be attributed to paramagnetic shift anisotropy due to the unpaired electrons. In the quantitative analysis of the ^{13}C NMR spectra, intensities of first- and second-order sidebands were added to the centerband intensity. DP spectra of nonprotonated and methyl carbons after recoupled dipolar dephasing by gated decoupling for 67 µs were also recorded.[3] The factors at which the signals of nonprotonated carbons were retained after 67-us recoupled dipolar dephasing were ≥ 0.9, as commonly observed. The measurement times for both types of DP spectra were 2.5 days for TMI-1, 3 days for TMI-2, and 1.7 days for TMI-3.

For determining structural contributions six chemical shift regions corresponding to main organic functional groups were defined and spectra were integrated accordingly (Table S3): 10 – 45 ppm (aliphatic carbons “alkyl-C”: -CH, $-CH_2$, and $-CH_3$ groups); 45 – 60 ppm (methoxy: $-OCH_3$ or N-substituted alkyl-C: NHC); 60 – 95 ppm (O-substituted aliphatic carbons: O-alkyl-C in such as alcohols, with minor ether -C-O- contributions); 95 – 105 ppm (O-C-O); 105 – 145 ppm (aromatic carbons; aryl-C); 145 – 164 ppm (phenolic carbons; O-aryl and aromatic CH_3 ethers); 164 – 185 ppm (carboxyl/amide/ester carbons: COO/N-C=O), and 185 – 220 ppm (quinone, aldehydes and ketones: C=O/HC=O). NMR spectra were also deconvolved using a molecular mixing model to estimate the content of biopolymer-like components such as carbohydrates, lignin, lipids, and charcoal (Table S4) as described by [4]. As all of these types of biopolymers contain carbonyl carbons (C=O) in their carboxyl (COO), aldehyde (CHO), or ketone (CO) functionalities, the contributions of carbonyl carbons were modeled and excluded as a separate category.

Table S3. Organic carbon speciation (%) in TMI HA using quantitative DP solid-state ^{13}C NMR.

	^{13}C NMR region (ppm)							
	**C=O/HC=O (185 – 220)**	**COO/N-C=O (164 – 185)**	**Aryl-O (145 – 164)**	**Aryl-C/H (105 – 145)**	**O-C-O (95 – 105)**	**O-alkyl-C (60 – 95)**	**OCH_3/NHC (45 – 60)**	**Alkyl-C (10 – 45)**
**TMI-1**	3.5	15.9	11.5	54.1	2.0	5.5	2.5	5.0
**TMI-2**	4.6	20.2	7.3	38.5	2.7	12.9	5.2	8.6
**TMI-3**	3.0	15.5	11.0	54.8	2.3	5.9	2.5	5.0

Table S4. Distribution of biomolecule-like compounds (% of C) in HA from deconvolution of solid-state ^{13}C NMR data.

	**Biomolecule class**					
	**Carbohydrate-like**	**Protein-like**	**Lignin-like**	**Lipid-like**	**Charcoal-like**	**Carbonyl-C (uncategorized)**
**TMI-1**	2.9	4.0	0.0	2.5	79.4	11.2
**TMI-2**	11.8	16.5	2.0	1.6	53.0	15.1
**TMI-3**	3.4	3.8	0.0	2.5	80.2	10.2

The integrals of the DP and DP/DD spectra were used to determine the fractions of non-protonated aromatic carbon (C- or O-substituted or interior to fused rings, labeled as $f_{arylCnp}$) and protonated aromatic carbon (labeled as $f_{arylC-H}$). First, $f_{arylCnp}$ was obtained by dividing the contribution from the 105 – 164 ppm region in the DP/DD spectra (non-protonated aromatic carbons) by the total area in the DP spectrum (all detected carbons). Subsequently, $f_{arylC-H}$ was calculated by difference (Eq. 2) from f_{arylC}, the latter being all detected aromatic carbons (105 – 164 ppm region in the DP spectrum).

$$f_{arylC-H} = f_{arylC} - f_{arylCnp} \quad \text{Eq. 2}$$

The fraction of carbons along the edges of condensed aromatic rings (χ_{edge}) was then calculated from $f_{arylC-H}$ (Eqs. 3 and 4). Edge carbons of condensed aromatic clusters include protonated aromatic (i.e., $f_{arylC-H}$) and oxygenated aromatic (e.g., phenolic, 145 – 164 ppm, labeled $f_{arylC-O}$) carbons collectively forming the minimum aromatic edge fraction ($\chi_{edge, min}$).[5]

$$\chi_{edge,min} = \chi_{C-H} + \chi_{C-O} \qquad \begin{cases} \chi_{C-H} = \dfrac{f_{arylC-H}}{f_{arylC}} \\ \chi_{C-O} = \dfrac{f_{arylC-O}}{f_{arylC}} \end{cases} \qquad \text{Eq. 3}$$

The maximum aromatic edge fraction also considers potential C=O and alkyl substitution of the aromatic rings:

$$\chi_{edge,max} = \chi_{C-H} + \chi_{C-O} + \chi_{C=O} + \chi_{alkyl} \qquad \text{Eq. 4}$$

with $\chi_{C=O} = \frac{(f_{C=O}+f_{COO/NC=O})}{f_{arylC}}$, see Table S5. The minimum number of carbons in the condensed aromatic cluster ($n_{C,min}$) was then calculated (Eq. 5):

$$n_{C,min} = \frac{6}{{\chi_{edge,max}}^2} \qquad \text{Eq. 5}$$

Subsequently, the number of condensed rings was systematically estimated using a linear regression on data from multiple polycondensed aromatic hydrocarbons (PAHs), correlating the number of carbon atoms with the number of aromatic rings in their structures (Fig. S1).

Table S5. Protonated fractions ($f_{arylC-H}$) and aromatic-referenced carbon fractions (χ), minimum and maximum aromatic edge fractions ($\chi_{edge,...}$), minimum number of carbons per aromatic cluster ($n_{C,min}$) and estimated minimum number of condensed aromatic rings (min. # rings) in HA samples.

	f_{arylC} (%)	$f_{arylCnp}$ (%)	$f_{arylC-H}$ (%)	χ_{C-H}	$\chi_{C=O}$	χ_{alkyl}	$\chi_{edge,min}$	$\chi_{edge,max}$	$n_{C,min}$	***min. # rings***
**TMI-1**	65.6	51.6	14.0	0.21	0.30	0.08	0.39	0.77	> 10 C	2
**TMI-2**	45.8	35.9	9.9	0.22	0.54	0.19	0.38	1.11	> 5 C	1
**TMI-3**	65.8	52.8	13.0	0.20	0.28	0.08	0.37	0.73	> 11 C	3

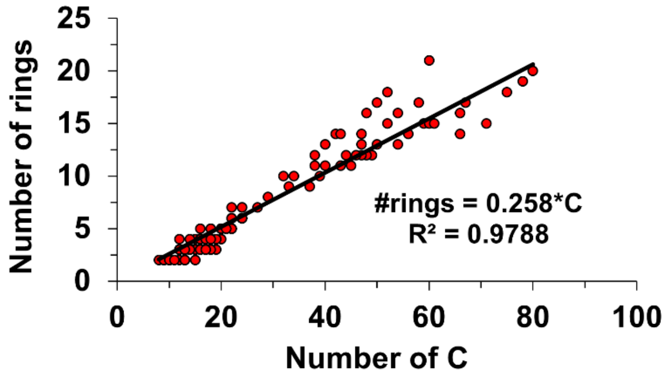

Fig. S1 Relationship between number of carbon atoms and number of rings in 193 PAHs.

Recoupled long-range $^{13}C\{^1H\}$ dephasing of nonprotonated carbons was performed at 7 kHz MAS with recoupling by composite ^1H 180° pulses to infer about the size of aromatic clusters.[6] Excitation by direct polarization after 1-, 5-s and 2-s recycle delays was used for TMI-1, TMI-2, and TMI-3, respectively. Spectra after long dephasing were measured with more scans and rescaled accordingly. Measurement times for the full series of data (40 µs to 2.57 ms) were 3 days each for TMI-1 and -3, while 3 days were also required for three data points from TMI-2 with its inferior signal-to-noise ratio.

Section 2.2. Solid-state ^{15}N NMR

Solid-state ^{15}N NMR was also performed on one of the HA samples (TMI-1) on a 400 MHz Bruker Advance II NMR spectrometer with a 4 mm HCN probe. A standard ramped-amplitude cross-polarization (ramp-CP) technique was used with a spinning speed of 14 kHz. Data acquisition parameters included 2,000,000 scans and a relaxation delay of 200 ms. $^{15}NH_4Cl$ was used for data quality control standard and chemical shift referencing (ammonium resonance at -341.15 ppm)[7].

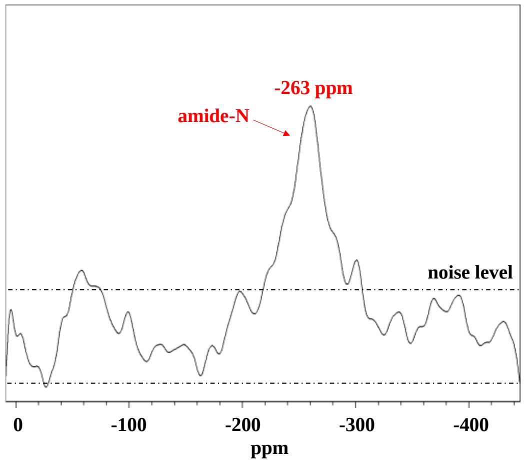

Fig. S2 Solid-state ^{15}N NMR spectrum of TMI-1.

Section 2.3. X-ray photoelectron spectroscopy (XPS)

XPS measurements were performed using a Thermo Scientific K-Alpha XPS. Survey spectra were measured with a 400 µm spatial resolution and 50.0 eV pass energies, resulting in a total acquisition time of 2 minutes and 23 seconds over 15 scans. An Al Kα X-ray gun source was used operating in standard lens and CAE analyzer modes. The energy step size was 0.100 eV across 191 energy steps. To further explore the speciation of bonds, high-resolution spectra of carbon, oxygen, and nitrogen were acquired and deconvolved using the Thermo Avantage software (version 5.957, ThermoFisher, Basingstoke, UK).

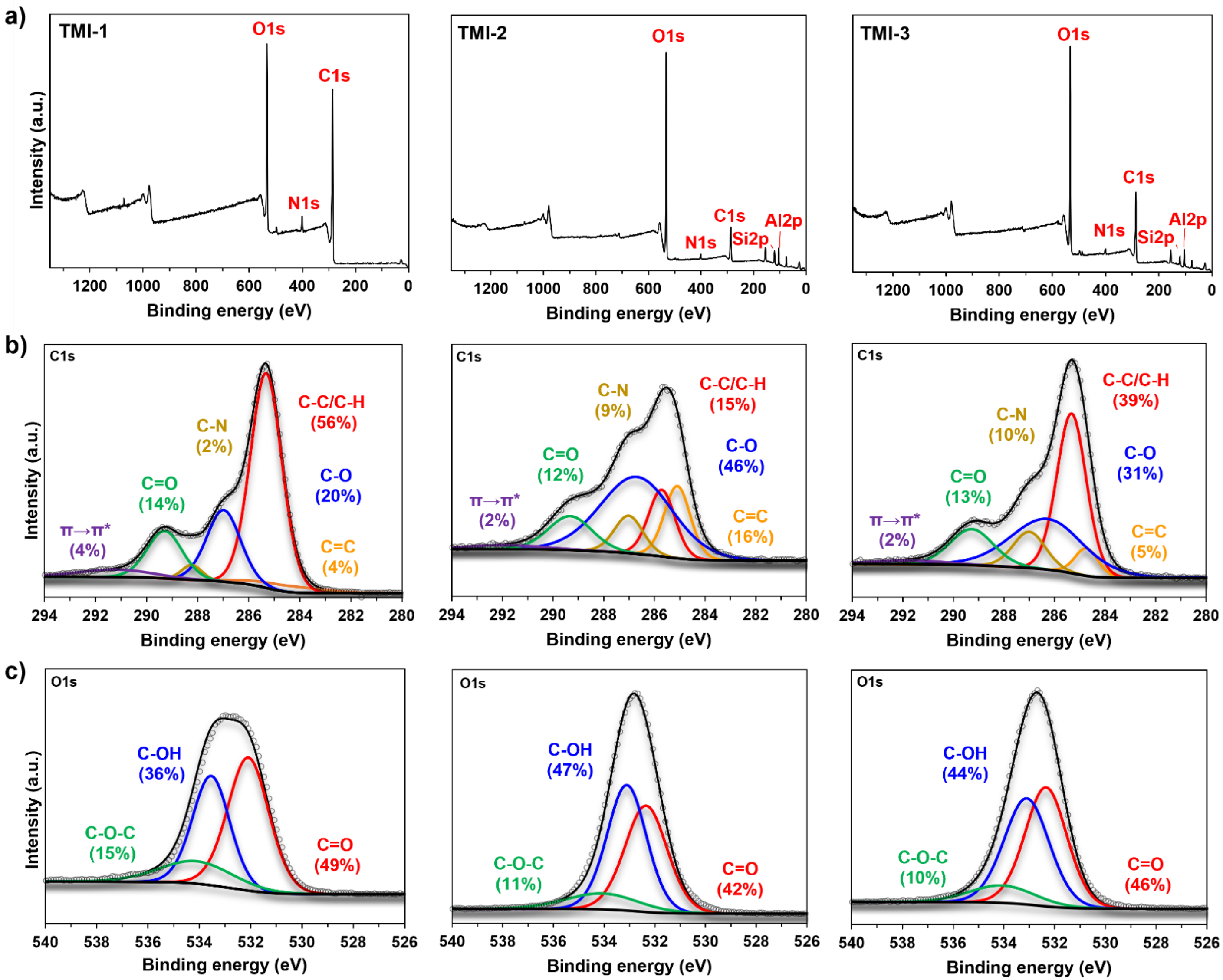

Fig. S3 X-ray photoelectron spectroscopy (XPS) of TMI HA. Survey spectra are shown on the top panels (a) with annotated peaks whereas high resolution carbon (C1s) and oxygen (O1s) spectra are shown as middle (b) and bottom panels (c), respectively.

Section 2.4. Raman spectroscopy

Raman spectra were recorded in triplicate using a circular laser line with a 532 nm excitation wavelength and a 50× objective lens to focus the laser on the sample surface. The measurements were taken with a 1% filter, 50 µm slit, 10 accumulations, and an acquisition time of 30 seconds using an XploRA Plus (Horiba, Japan). The G band and D bands were deconvolved to calculate the D-to-G intensity ratio (I_D/I_G). The deconvolution also yielded values for the full width at half maximum for peak D (FWHM-D). The I_D/I_G ratio and FWHM-D were used as indicators for carbon network disorder following most recent advancements in Raman spectroscopy.[8–11]

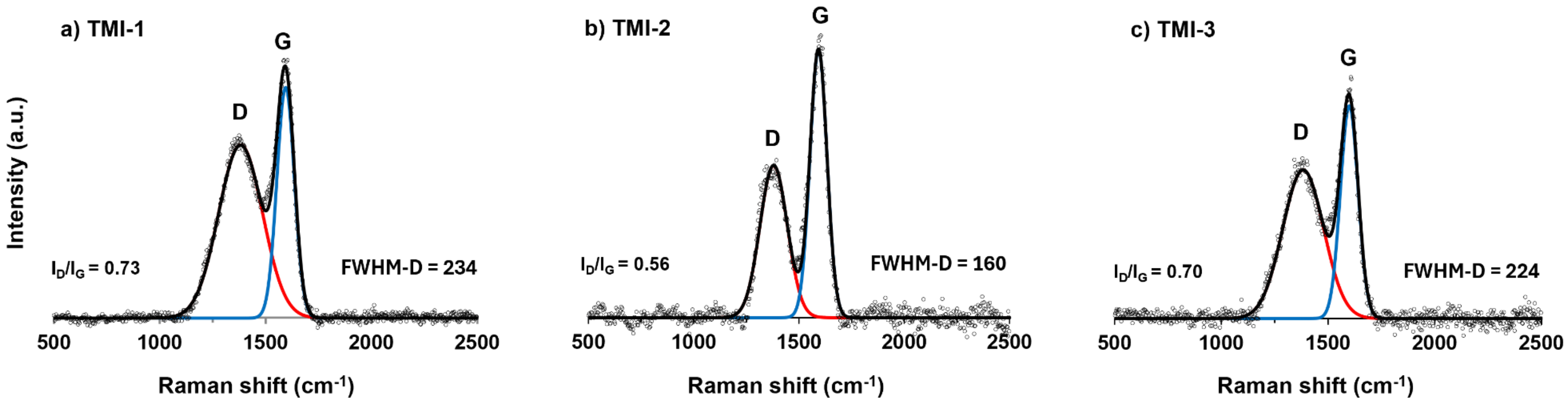

Figure S4. Raman spectra of TMI humic acid samples. Shown are the raw spectral data (circles) along with fitted D (red) and G (blue) vibrational bands, the total deconvolved spectrum (black line), as well as the calculated D-to-G ratio (I_D/I_G) and full width at half maximum of the D peak (FWHM-D).

Section 3. Molecular fingerprinting using ultrahigh resolution mass spectrometry (ESI-FT-ICR-MS)

Section 3.1. Instrumental Analysis

Powdered HA samples were re-dissolved in 0.1 M NaOH and sonicated for 10 min. They were then treated with a Dowex TM 50WX8-100 ion-exchange resin to remove sodium and other inorganic ions that interfere with the ESI. The supernatant was combined with methanol to yield a 1:1 methanol:water solution at a final sample-carbon concentration of 50 mg-C L^{-1} following the $CE_{EXTRACTION}$

protocol described by [12]. The samples were introduced at a rate of 120 µL hr^{-1} into an Apollo II ESI source (negative-ion mode) coupled to a 10-Tesla Bruker Daltonics Apex Qe FT-ICR-MS. Prior to analysis, the instrument was externally calibrated using polyethylene glycol[12]. Suwannee River fulvic acid standard from the International Humic Substances Society was used for tune validation and data quality control.[13] TMI HA samples were analyzed across a mass range of 200 – 800 Da for a total of 300 scans. Procedural blanks were run to ensure the absence of carryover effects. ESI voltages and ion accumulation delays were optimized for each sample to guarantee consistent spraying and comparable number of detected ions for each sample. The analytical reproducibility of the FT-ICR-MS measurements is within a relative standard deviation of 10 %.[14]

Section 3.2. Data Analysis

After mass spectra were acquired, ions with signal-to-noise ratio ≥ 3 were peak-picked. Subsequently, mass lists were internally calibrated with naturally abundant fatty acids, dicarboxylic acids, and CH_2 homologous series.[15] Peaks present in process blanks, as well as salts, doubly charged, and isotopologue (^{13}C) peaks were removed. Remaining ion peaks were assigned molecular formulas with elemental ranges of $^{12}C_{5-\infty}$, $^{1}H_{5-100}$, $^{16}O_{1-30}$, $^{14}N_{0-5}$, $^{32}S_{0-4}$, and $^{31}P_{0-2}$. Ambiguous peak assignments were eliminated by homologous series refinement (CH_2, H_2, COO, CH_2O, O_2, H_2O, NH_3) [16] and the final formula lists contained formulas within ± 1 ppm error. All data analysis was done using the MATLAB-based Toolbox for Environmental Research "TEnvR" [17].

The nominal oxidation state of carbon (NOSC) and the modified aromaticity index (AI_{mod})[18] were calculated for all formulas. Molecular formulas were then classified as following: condensed aromatic compounds (ConAC, $AI_{mod} \geq 0.67$, number of C-atoms ≥ 15), aromatic compounds with aliphatic side chains ($0.67 < AI_{mod} \leq 0.50$), olefinic/alicyclic compounds ($0 < AI_{mod} < 0.50$), and aliphatic compounds ($AI_{mod} = 0$). Molecular formulas were grouped in two additional compound classes based on their molecular properties: carboxyl-containing aliphatic molecules (CCAM) ($0.85 \leq H/C \leq 2$, $O/C \leq 0.4$) and lignin ($0.2 \leq O/C \leq 0.6$; $0.6 \leq H/C \leq 1.2$). Lastly, molecular formulas were categorized based on the presence of non-O heteroelements: CHO, CHON, CHOS, and CHOP compounds[19].

Kendrick mass defect (KMD) analysis was used for assessing homologous series[20]. KMD values were calculated for carboxyl (COO) and ammonia (NH_3) series (S). The Kendrick mass (KM) was first calculated using the molecular weight of each compound (Eq. 5). The Kendrick nominal mass (KNM) was then determined as the integer of KM. The KMD was calculated as the difference between KM and KNM (Eq. 6).

$$KM = \text{Molecular Weight} \times S$$
$$\text{Where } S = \frac{44.0000000}{43.9898292} \text{ for COO series; } = \frac{17.000000}{17.026549} \text{ for } NH_3 \text{ series;} \quad \text{Eq. 5}$$

$$KMD = KM - KNM \text{ (integer of KM)} \quad \text{Eq. 6}$$

Section 3.3. Results

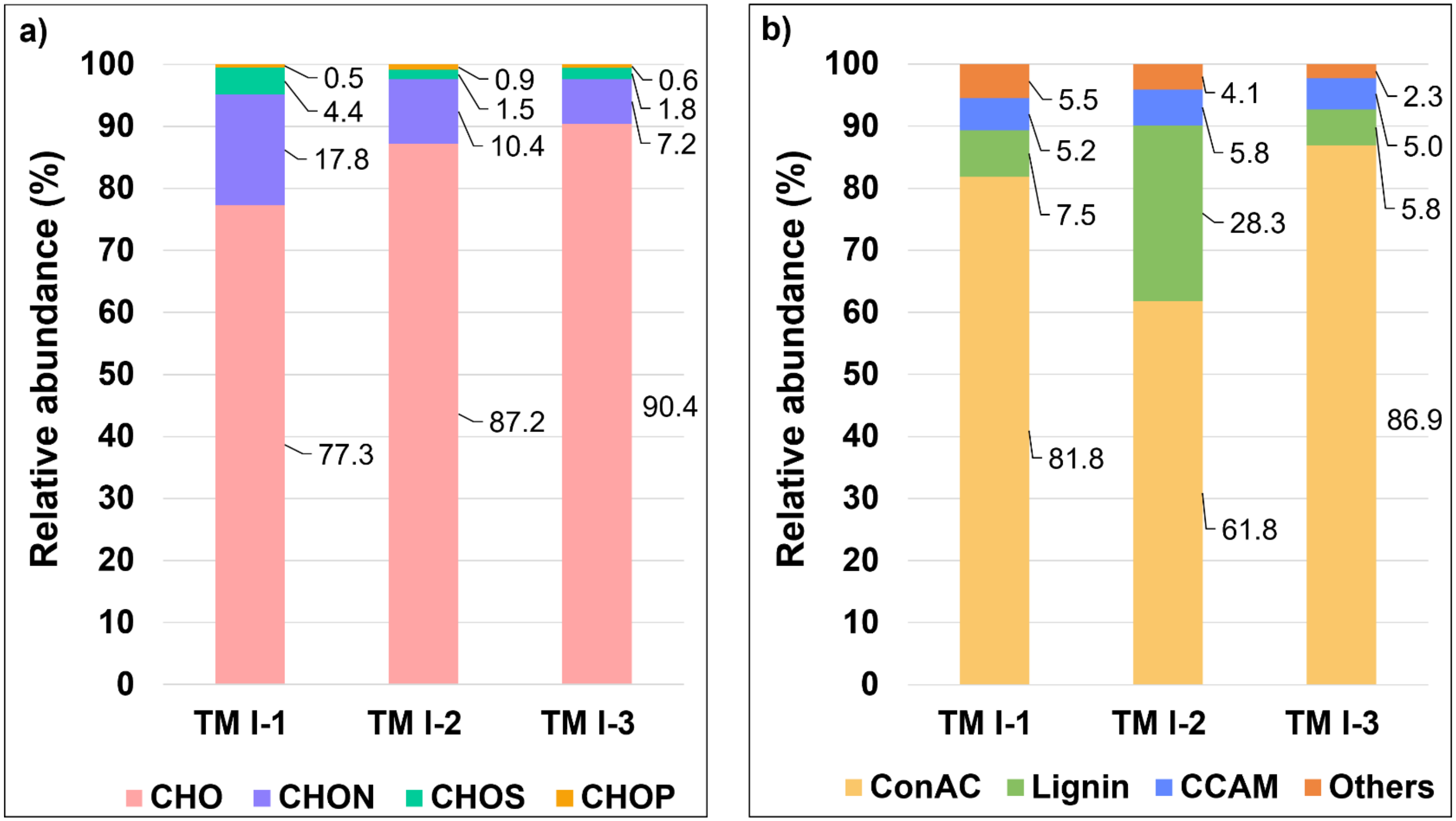

Fig. S5 Relative abundance (%) of molecular formulas (a) and biochemical classes (b) in the three HA samples. ConAC = Condensed aromatic carbon; CCAM = carboxyl containing aliphatic molecules, which represents the sum of lipids + proteins.

Table S6. Composition of condensed aromatic carbon (ConAC) molecular formulas.

Sample	Contribution (%)			
	CHO	CHON	CHOS	CHOP
**TMI-1**	80.6 ± 4.0	19.2 ± 1.0	0.2 ± 0.0	0.0 ± 0.0
**TMI-2**	90.3 ± 4.5	9.6 ± 0.5	0.1 ± 0.0	0.0 ± 0.0
**TMI-3**	89.7 ± 4.5	10.3 ± 0.5	0.0 ± 0.0	0.0 ± 0.0

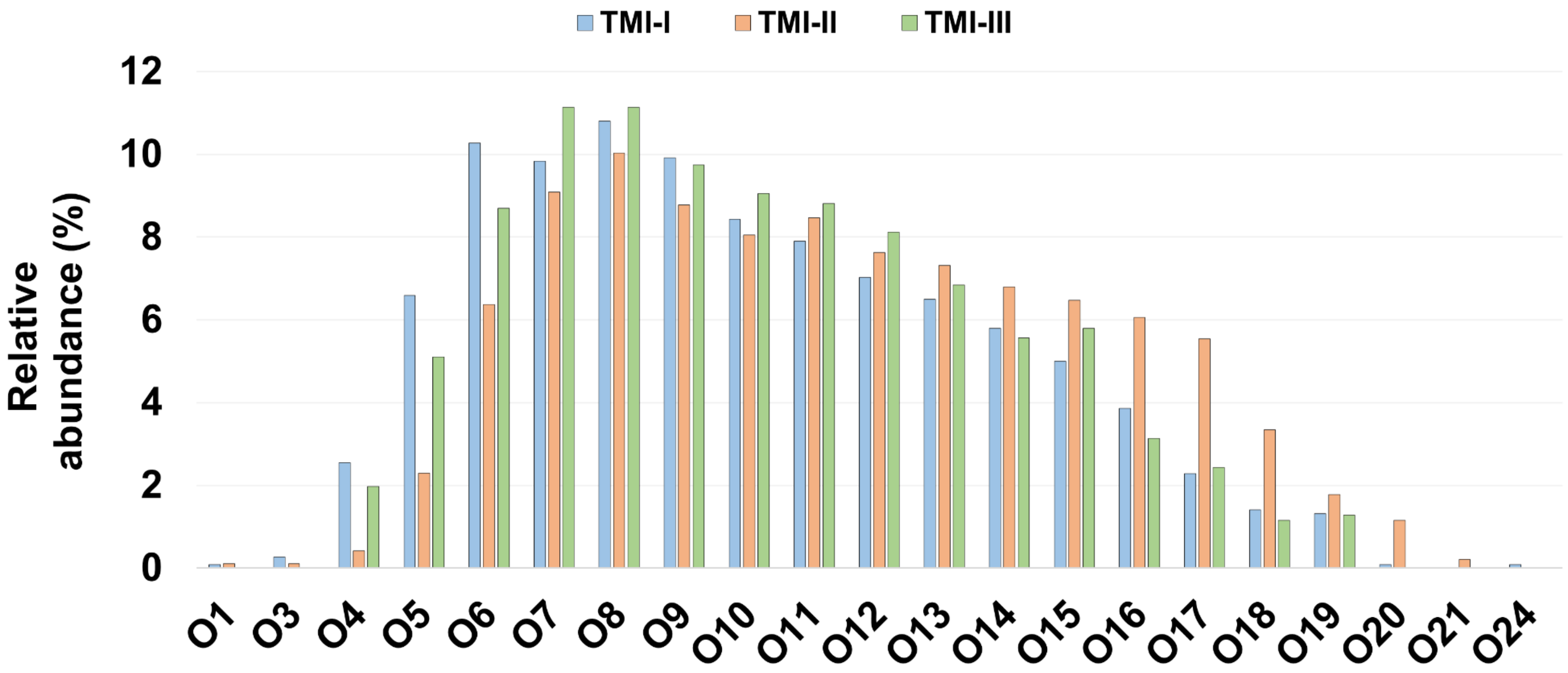

Fig. S6 Distribution of oxygen classes (O_x, x = 1 – 24) for ConAC molecular formulas.

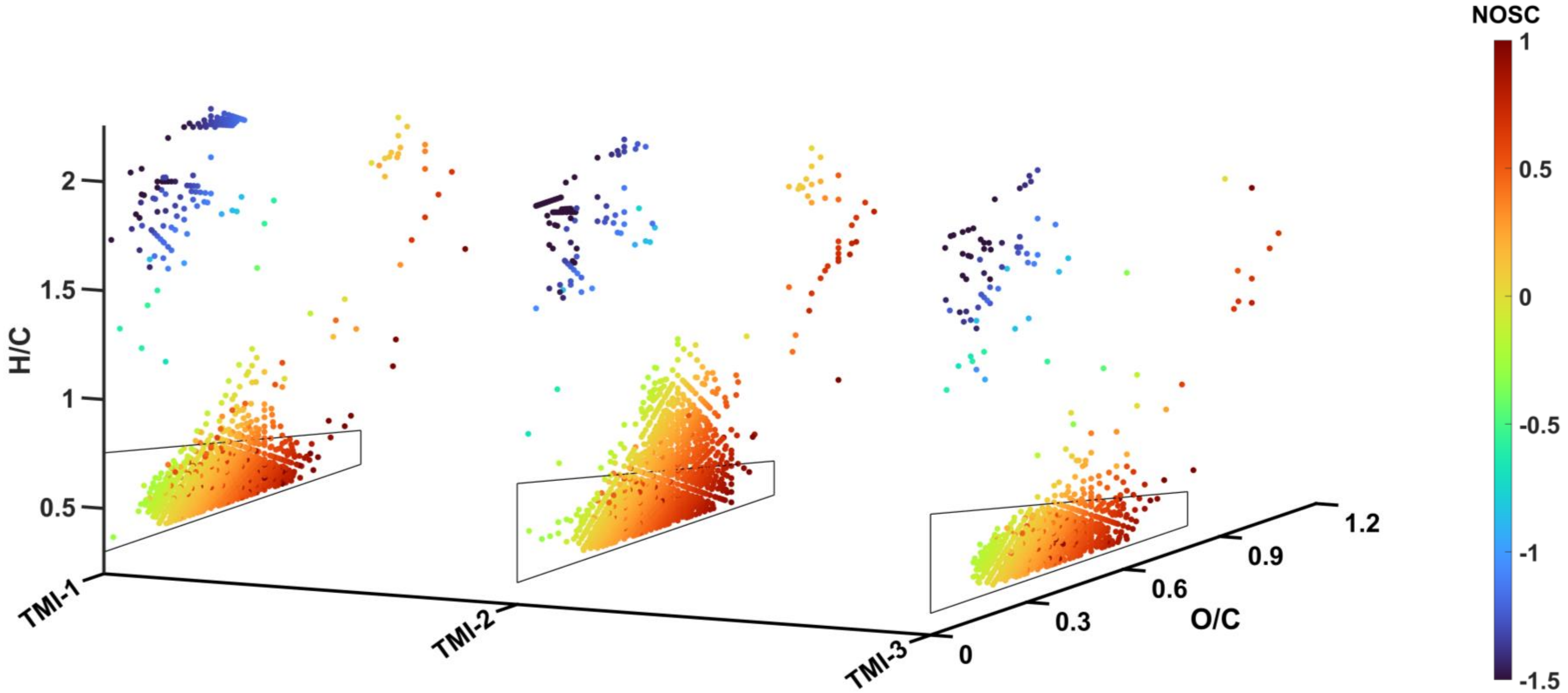

Fig. S7. Van Krevelen diagrams of humic acids with molecular formulas color-coded based on nominal oxidation state of carbon (NOSC). The boxed area represents the ConAC region ($AI_{MOD} \geq 0.67$).

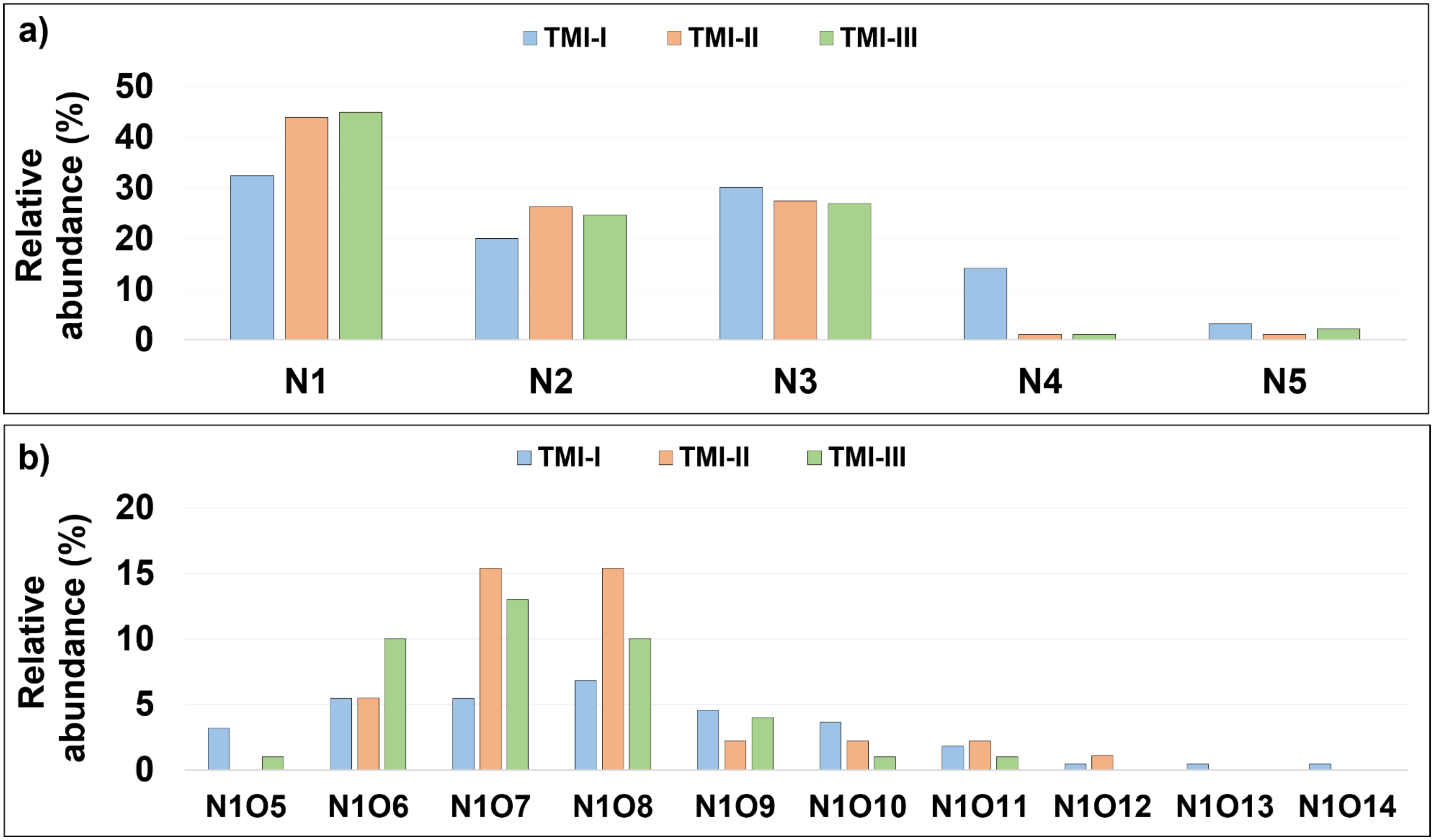

Fig. S8 Distribution of nitrogen classes (N_x, $x = 1 – 5$, top panel) and oxygen classes containing one nitrogen (N_1O_x, $x = 5 – 14$) in ConAC molecular formulas.

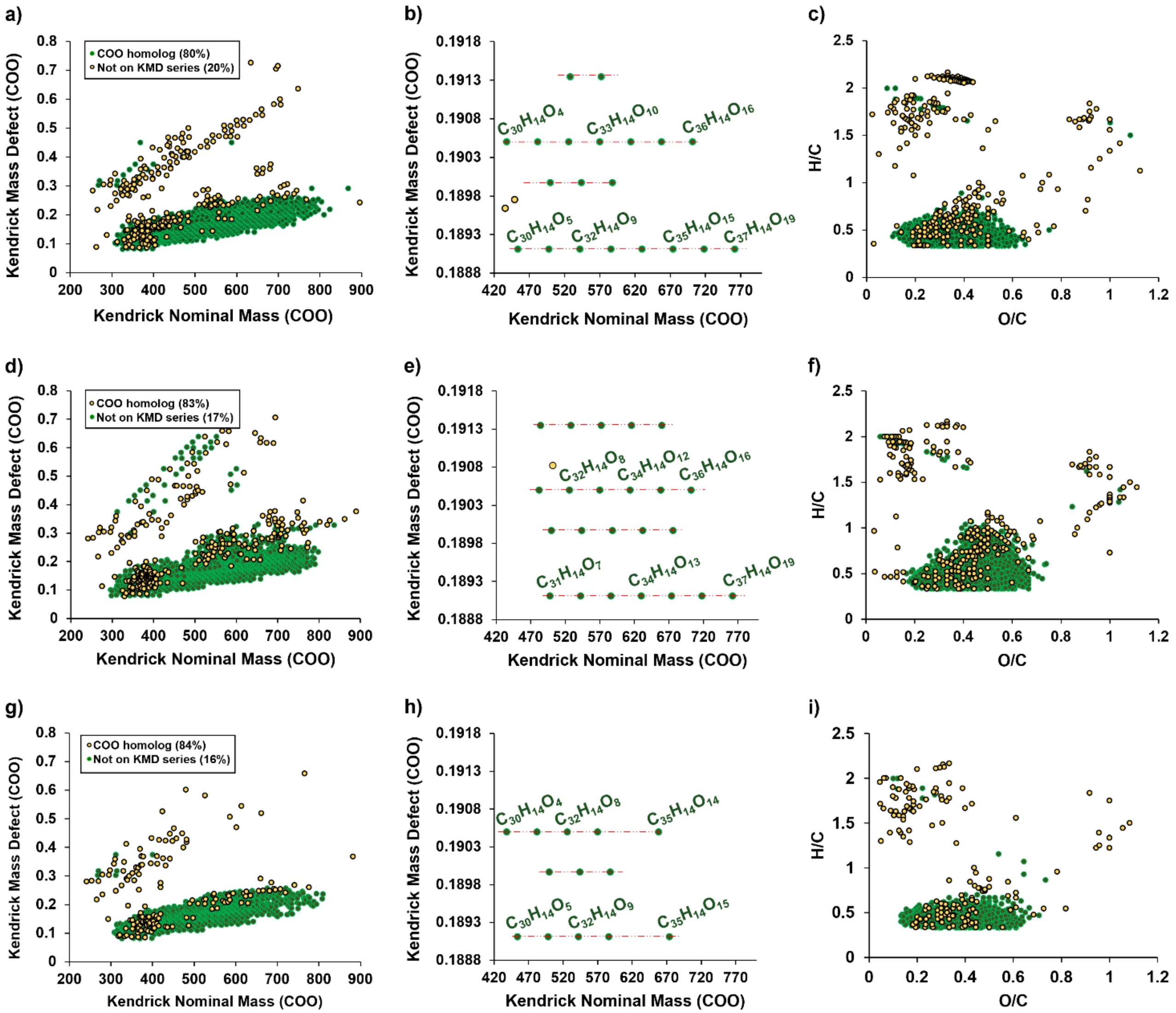

Fig S9. Kendrick mass defect (KMD) analysis using carboxyl series of formulas of TMI-1 (a-c), TMI-2 (d-f), and TMI-3 (g-i) HA. Formulas in green represent species potentially involved in oxygenation reactions. Formulas that were not part of KMD series are colored in yellow. Panels (a,d,g) shows the whole KMD plots while panels (b,e,h) show expanded regions. For clarity, only the molecular formulas for one of the series for each plot are labeled. Panels (c,f,i) show the van Krevelen distributions of the formulas.

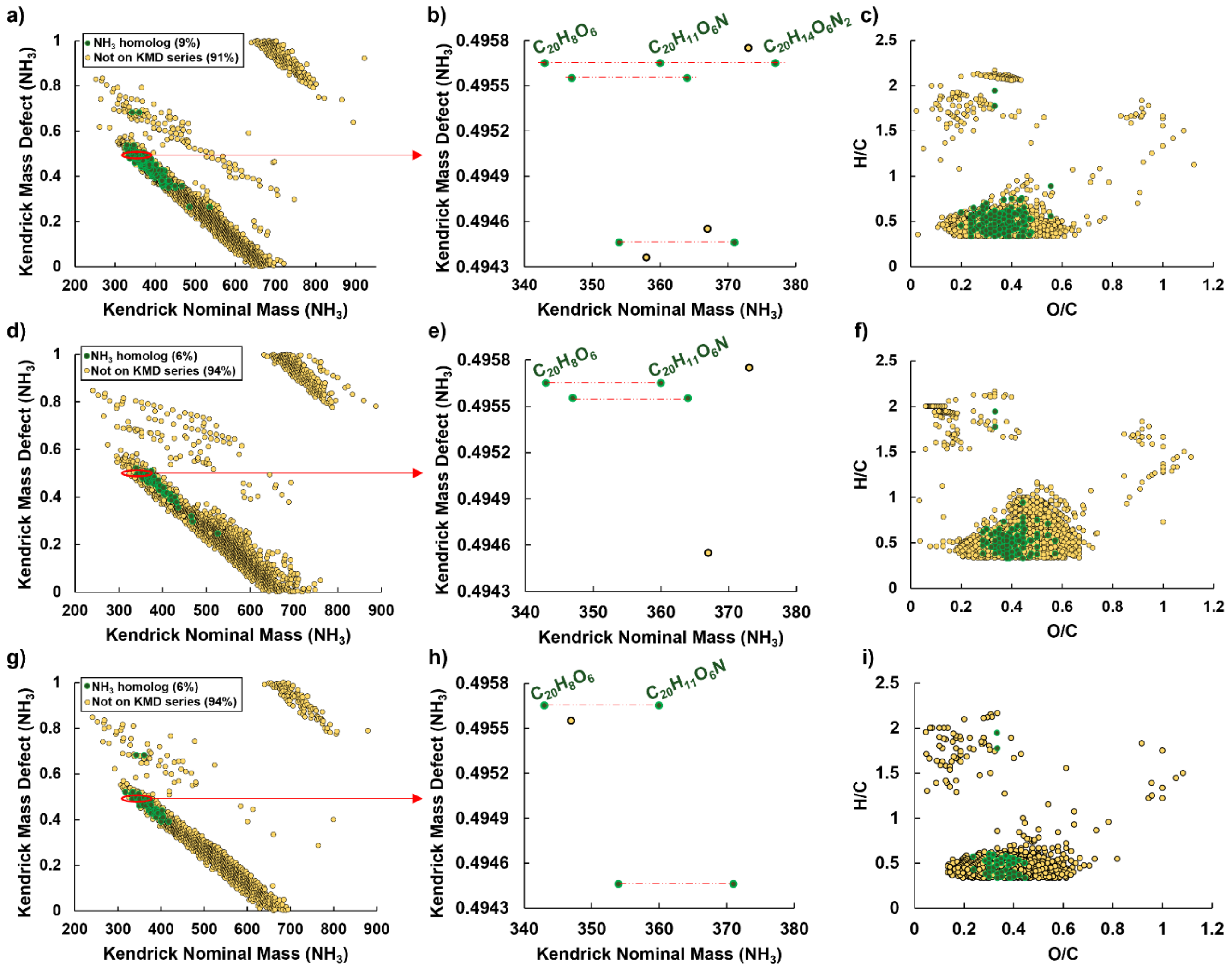

Fig S10. Kendrick mass defect (KMD) analysis using ammonia series of formulas of TMI-1 (a-c), TMI-2 (d-f), and TMI-3 (g-i) HA. Formulas in green represent species potentially involved in ammonia reactions. Formulas that were not part of KMD series are colored in yellow. Panels (a,d,g) shows the whole KMD plots while panels (b,e,h) show expanded regions. For clarity, only the molecular formulas for one of the series for each plot are labeled. Panels (c,f,i) shows the van Krevelen distributions of the formulas.

Section 4. Quantification of condensed aromatic carbon (ConAC) and nitrogen (ConAN)

Section 4.1. Benzenepolycarboxylic acid (BPCA) analysis

Dried powdered HA samples (2 – 10 mg carbon-equivalents) were weighed into pre-combusted vials and were dissolved in 1 mL of ultrapure water (18.2 MΩ) amended with NH_4OH (Fluka, ≥25 wt %, LC-MS grade) to pH 9 [12] to ensure complete HA solubilization. Each solution was pipetted in two 0.5 mL vials for duplicate analysis. The solvent was evaporated at 60 °C after which concentrated nitric acid (0.5 mL, 65% HNO_3, J.T. Baker, trace metal grade) was added and ampules were allowed to sit for 15 min to allow any produced gasses to escape[21]. Ampules were flame sealed and thermolyzed in a programmable oven for 6 hours at 160 °C. No more than 5 mg carbon-equivalents were digested to avoid artificial production of BPCA molecules[22]. After the digestion, the nitric acid was evaporated at 60 °C in a sand bath under a gentle stream of ultrapure N_2 gas (Airgas, UHP300). The BPCA-containing residue was then dissolved in 1 mL of 0.6 M phosphoric acid and filtered using a 0.2 µm PTFE filter into an autosampler vial.

The produced BPCA markers range from highly substituted benzenehexa- (B6CA) and benzenepentacarboxylic (B5CA) acids to less substituted benzenedi-, benzenetri-, and benzenetetracarboxylic acids (B2CA, B3CA, B4CA, respectively) and their nitrated derivatives[23]. The B2CA, B3CA, and B4CA are generally of low yield[24] and can be produced from non-condensed molecules such as lignin[22,25]. Thus, only the concentrations of B6CA and B5CA were used to estimate the ConAC content.

BPCA markers were quantified using high performance liquid chromatography with spectrophotometric detection at 254 nm on an Agilent 1100 system. Separation was achieved using organic-free eluents of 0.6 M phosphoric acid (pH = 1) and phosphate buffer (20 mM, pH = 6) on an Agilent Poroshell 120 Phenyl-Hexyl (4.6 x 150 mm, 2.7 µm) column with conditions described previously by [21]. B6CA and B5CA markers were quantified using external calibration curves of commercially available B6CA and B5CA standards (Aldrich, > 99% purity). The two technical replicates of each HA yielded relative standard deviations below 5%. The measured B6CA and B5CA quantities in carbon-equivalents were used to estimate the concentration of ConAC in each sample using the scaling factor of 7.04 proposed by [25], i.e., $ConAC_{BPCA}$ (Eq. 7). The quantified ConAC (nominator) is then presented relative to the organic carbon amount in the sample before digestion (denominator).

$$\mathrm{ConAC_{BPCA}}\ (\%) = \frac{7.04\ \times [\mathrm{B6CA_C(mg)} + \mathrm{B5CA_C(mg)}]}{\mathrm{Sample\ weight\ (mg)}\ \times \mathrm{C\%}} \times 100 \qquad \text{Eq. 7}$$

Larger ConAC structures produce more B6CA relative to B5CA[23,26–28] and thus, the molar ratio of B6CA to B5CA (Eq. 8) serves as a proxy for the average size of ConAC clusters (i.e., degree of condensation of ConAC), with higher B6CA:B5CA ratio corresponding to ConAC molecules of larger cluster size.

$$\mathrm{B6CA{:}\,B5CA\ ratio} = \frac{\mathrm{mol\ B6CA\ produced}}{\mathrm{mol\ B5CA\ produced}} \qquad \text{Eq. 8}$$

Raw BPCA data (quantities of BPCA markers in carbon-equivalents produced during the HNO_3 oxidation, i.e., mg $B5CA_C$ or $B6CA_C$ per mg sample-carbon) are provided in Table S7 along with calculated $ConAC_{BPCA}$ contents and B6CA:B5CA ratios.

Table S7. Quantities of benzenehexa- (B6CA) and benzenepentacarboxylic acid (B5CA) molecular markers after HNO_3 oxidation and calculated condensed aromatic carbon ($ConAC_{BPCA}$) quantities.

	**$B6CA_C$/$Sample_C$ x1000**	**$B5CA_C$/$Sample_C$ x1000**	**$ConAC_{BPCA}$ (%)**	**Average ± St. Dev**	**B6CA:B5CA ratio**	**Average ± St. Dev**
**TMI-1**	22.37	31.28	37.77	37.74 ± 0.04	0.656	0.663 ± 0.011
	22.64	30.93	37.72		0.671	
**TMI-2**	3.67	5.03	6.13	5.99 ± 0.20	0.670	0.684 ± 0.020
	3.59	4.72	5.85		0.698	
**TMI-3**	19.40	25.65	31.71	30.93 ± 1.11	0.693	0.691 ± 0.003
	18.38	24.44	30.14		0.689	

Section 4.2. Chemothermal oxidation (CTO) analysis

Dried powdered HA samples (0.8 – 1.9 mg) in triplicate were weighed into pre-combusted silver capsules (COSTECH, 3.5 x 5 mm) and then thermolyzed in oxic conditions at 375 ^{O}C for 18 hours[29]. The residue in the capsules was then treated with HCl to remove any carbonates that could have formed during the thermolysis. The remaining organic residue is assumed to be the ConAC/ConAN fraction that this method detects ($ConAC_{CTO}$ and $ConAN_{CTO}$, respectively). The capsules were then analyzed on a Thermo Finnigan FlashEA 1112 elemental analyzer fitted with a standard CHN column for determining the carbon (C%) and nitrogen (N%) contents of the residues. Data were calibrated to a five-point external calibration curve of nicotinamide (CE Elantech, Inc.). Empty silver capsules were analyzed as blanks to evaluate for any sample carryover or other background contamination. An aspartic acid standard (CE Elantech, Inc.) was also analyzed as a control sample to confirm the accuracy of the measurements. ConAC and ConAN were calculated by multiplying the weights of material in the capsule before (HA) and after thermolysis (residue) with the corresponding elemental percentage (Eqs. 9 and 10, respectively).

$$ConAC_{CTO}\% = \frac{Residue\ weight \times C\%_{residue}}{HA\ weight \times C\%_{HA}} \quad \text{Eq. 9}$$

$$ConAN_{CTO}\% = \frac{Residue\ weight \times N\%_{residue}}{HA\ weight \times N\%_{HA}} \quad \text{Eq. 10}$$

Table S8. Quantities of condensed aromatic carbon measured via chemothermal oxidation ($ConAC_{CTO}$). (*) from dos Santos et al. (2020).

	HA (mg)	C% of HA(*)	Residue (mg)	C % of residue	$ConAC_{CTO}$ (%)	Average ± St. Dev
**TMI-1**	1.445	51.240	0.781	10.195	10.75	12.6 ± 2.3
	1.874		1.032	11.048	11.87	
	1.688		0.988	13.212	15.09	
**TMI-2**	1.894	39.620	1.475	0.580	1.14	0.8 ± 0.3
	1.416		1.097	0.289	0.56	
	1.356		1.056	0.398	0.78	
**TMI-3**	0.880	50.220	0.612	47.411	65.66	59.5 ± 5.5
	0.926		0.567	44.995	54.86	
	1.229		0.779	45.979	58.03	

Table S9. Quantities of condensed aromatic nitrogen measured via chemothermal oxidation ($ConAN_{CTO}$). (*) from dos Santos et al. (2020).

	HA (mg)	N% of HA(*)	Residue (mg)	N % of residue	$ConAN_{CTO}$ (%)	Average ± St. Dev
**TMI-1**	1.445	3.410	0.781	0.782	12.40	14.1 ± 2.2
	1.874		1.032	0.822	13.27	
	1.688		0.988	0.967	16.60	
**TMI-2**	1.894	4.040	1.475	0.132	2.55	2.3 ± 0.3
	1.416		1.097	0.125	2.40	
	1.356		1.056	0.099	1.90	
**TMI-3**	0.880	2.970	0.612	2.812	65.84	61.1 ± 4.6
	0.926		0.567	2.749	56.67	
	1.229		0.779	2.855	60.92	

Section 5. Characteristics of ConAC and ConAN structures

Table S10. Structural characteristics of condensed aromatic carbon (ConAC) compounds including number of rings determined by electrospray ionization – Fourier transform – ion cyclotron resonance – mass spectrometry (ESI-FT-ICR-MS), direct polarization (DP) or recoupled long-range $^{13}C\{^{1}H\}$ dephasing solid-state ^{13}C NMR, and quantification of benzenehexa- (B6CA) and benzenepentacarboxylic acid (B5CA) markers.

	H/C ratio from EA	H/C ratio of ConAC formulas from ESI-FT-ICR-MS	# of rings from ESI-FT-ICR-MS	Minimum number of rings from DP NMR	Number of rings from recoupled long-range dephasing	B6CA:B5CA ratio
**TMI-1**	0.89 ± 0.02	0.45 ± 0.07	8	2	6 – 8	0.663 ± 0.01
**TMI-2**	1.23 ± 0.06	0.45 ± 0.07	8	1	6 – 8	0.684 ± 0.02
**TMI-3**	0.88 ± 0.00	0.45 ± 0.07	8	3	6 – 8	0.691 ± 0.00

Table S11. C/N ratios for condensed aromatic nitrogen (ConAN) determined using chemothermal oxidation (CTO), X-ray photoemission spectroscopy (XPS) and electrospray ionization – Fourier transform – ion cyclotron resonance – mass spectrometry (FT-ICR-MS) methodologies.

	C/N from EA on whole samples (mol/mol)	C/N from XPS on whole samples (mol/mol)	C/N of all formulas from ESI-FT-ICR-MS (mol/mol)	C/N from EA on CTO residue (mol/mol)	C/N of ConAN formulas from ESI-FT-ICR-MS (mol/mol)
**TMI-1**	17.80 ± 0.68	17.8 ± 0.9	11.09 ± 6.40	15.61 ± 0.37	11.17 ± 6.50
**TMI-2**	11.38 ± 0.18	13.2 ± 0.7	13.04 ± 6.20	4.17 ± 1.30	13.06 ± 6.13
**TMI-3**	19.95 ± 0.20	16.2 ± 0.8	12.56 ± 6.50	19.18 ± 0.45	13.26 ± 6.35

References

(1) EMBRAPA. *Manual de Metodos de Análise de Solo*; Centro Nacional de Pesquisa de Solos, 1997.

(2) ASTM. *Standard Test Methods for Moisture, Ash, and Organic Matter of Peat and Other Organic Soils*; West Conshohocken, 2014. https://doi.org/10.1520/D2974-14.

(3) Mao, J. D.; Schmidt-Rohr, K. Accurate Quantification of Aromaticity and Nonprotonated Aromatic Carbon Fraction in Natural Organic Matter by 13C Solid-State Nuclear Magnetic Resonance. *Environ Sci Technol* **2004**, *38* (9), 2680–2684. https://doi.org/10.1021/es034770x.

(4) Nelson, P. N.; Baldock, J. A. Estimating the Molecular Composition of a Diverse Range of Natural Organic Materials from Solid-State 13C NMR and Elemental Analyses. *Biogeochemistry* **2005**, *72* (1), 1–34. https://doi.org/10.1007/s10533-004-0076-3.

(5) Brewer, C. E.; Schmidt-Rohr, K.; Satrio, J. A.; Brown, R. C. Characterization of Biochar from Fast Pyrolysis and Gasification Systems. *Environ Prog Sustain Energy* **2009**, *28* (3), 386–396. https://doi.org/10.1002/ep.10378.

(6) Mao, J. D.; Schmidt-Rohr, K. Recoupled Long-Range C-H Dipolar Dephasing in Solid-State NMR, and Its Use for Spectral Selection of Fused Aromatic Rings. *Journal of Magnetic Resonance* **2003**, *162* (1), 217–227. https://doi.org/10.1016/S1090-7807(03)00012-0.

(7) Wei, J.; Knicker, H.; Zhou, Z.; Eckhardt, K. U.; Leinweber, P.; Wissel, H.; Yuan, W.; Brüggemann, N. Nitrogen Immobilization Caused by Chemical Formation of Black- and Amide-N in Soil. *Geoderma* **2023**, *429*. https://doi.org/10.1016/j.geoderma.2022.116274.

(8) Fregolente, L. G.; Rodrigues, M. T.; Oliveira, N. C.; Araújo, B. S.; Nascimento, Í. V.; Souza Filho, A. G.; Paula, A. J.; Costa, M. C. G.; Mota, J. C. A.; Ferreira, O. P. Effects of Chemical Aging on Carbonaceous Materials: Stability of Water-Dispersible Colloids and Their Influence on the Aggregation of Natural-Soil Colloid. *Science of the Total Environment* **2023**, *903*. https://doi.org/10.1016/j.scitotenv.2023.166835.

(9) Senneca, O.; Apicella, B.; Russo, C.; Cerciello, F.; Salatino, P.; Heuer, S.; Wütscher, A.; Schiemann, M.; Muhler, M.; Scherer, V. Pyrolysis and Thermal Annealing of Coal and Biomass in CO2-Rich Atmospheres. *Energy and Fuels* **2018**, *32* (10), 10701–10708. https://doi.org/10.1021/acs.energyfuels.8b02417.

(10) Phounglamcheik, A.; Wang, L.; Romar, H.; Kienzl, N.; Broström, M.; Ramser, K.; Skreiberg, Ø.; Umeki, K. Effects of Pyrolysis Conditions and Feedstocks on the Properties and Gasification Reactivity of Charcoal from Woodchips. *Energy and Fuels* **2020**, *34* (7), 8353–8365. https://doi.org/10.1021/acs.energyfuels.0c00592.

(11) Makowska, M.; Dziosa, K. Influence of Different Pyrolysis Temperatures on Chemical Composition and Graphite-like Structure of Biochar Produced from Biomass of Green Microalgae Chlorella Sp. *Environ Technol Innov* **2024**, *35*. https://doi.org/10.1016/j.eti.2024.103667.

(12) Goranov, A. I.; Tadini, A. M.; Martin-Neto, L.; Bernardi, A. C. C.; Oliveira, P. P. A.; Pezzopane, J. R. M.; Milori, D. M. B. P.; Mounier, S.; Hatcher, P. G. Comparison of Sample Preparation Techniques for the (-)ESI-FT-ICR-MS Analysis of Humic and Fulvic Acids. *Environ Sci Technol* **2022**, *56* (17), 12688–12701. https://doi.org/10.1021/acs.est.2c01125.

(13) Hawkes, J. A.; D'Andrilli, J.; Agar, J. N.; Barrow, M. P.; Berg, S. M.; Catalán, N.; Chen, H.; Chu, R. K.; Cole, R. B.; Dittmar, T.; Gavard, R.; Gleixner, G.; Hatcher, P. G.; He, C.; Hess, N. J.; Hutchins, R. H. S.; Ijaz, A.; Jones, H. E.; Kew, W.; Khaksari, M.; Palacio Lozano, D. C.; Lv, J.; Mazzoleni, L. R.; Noriega-Ortega, B. E.; Osterholz, H.; Radoman, N.; Remucal, C. K.; Schmitt, N. D.; Schum, S. K.; Shi, Q.; Simon, C.; Singer, G.; Sleighter, R. L.; Stubbins, A.; Thomas, M. J.; Tolic, N.; Zhang, S.; Zito, P.; Podgorski, D. C. An International Laboratory Comparison of Dissolved Organic Matter Composition by High Resolution Mass Spectrometry: Are We Getting the Same Answer? *Limnol Oceanogr Methods* **2020**, *18* (6), 235–258. https://doi.org/10.1002/lom3.10364.

(14) Sleighter, R. L.; Chen, H.; Wozniak, A. S.; Willoughby, A. S.; Caricasole, P.; Hatcher, P. G. Establishing a Measure of Reproducibility of Ultrahigh-Resolution Mass Spectra for Complex Mixtures of Natural Organic Matter. *Anal Chem* **2012**, *84* (21), 9184–9191. https://doi.org/10.1021/ac3018026.

(15) Sleighter, R. L.; Hatcher, P. G. Molecular Characterization of Dissolved Organic Matter (DOM) along a River to Ocean Transect of the Lower Chesapeake Bay by Ultrahigh Resolution Electrospray Ionization Fourier Transform Ion Cyclotron Resonance Mass Spectrometry. *Mar Chem* **2008**, *110* (3–4), 140–152. https://doi.org/10.1016/j.marchem.2008.04.008.

(16) Koch, B. P.; Dittmar, T.; Witt, M.; Kattner, G. Fundamentals of Molecular Formula Assignment to Ultrahigh Resolution Mass Data of Natural Organic Matter. *Anal Chem* **2007**, *79* (4), 1758–1763. https://doi.org/10.1021/ac061949s.

(17) Goranov, A. I.; Sleighter, R. L.; Yordanov, D. A.; Hatcher, P. TEnvR: MATLAB-Based Toolbox for Environmental Research. *Analytical Methods* **2023**, *15* (40), 5390–5400. https://doi.org/10.1039/D3AY00750B.

(18) Koch, B. P.; Dittmar, T. From Mass to Structure: An Aromaticity Index for High-Resolution Mass Data of Natural Organic Matter. *Rapid Communications in Mass Spectrometry* **2006**, *20* (5), 926–932. https://doi.org/10.1002/rcm.2386.

(19) dos Santos, J. V.; Goranov, A. I.; Bento, L. R.; Oliveira, P. P. A.; Pezzopane, J. R. M.; Bernardi, A. C. C.; de Sá, Í. P.; Nogueira, A. R. A.; Martin-Neto, L.; Hatcher, P. G. Biogeochemistry of Dissolved Organic Matter and Inorganic Solutes in Soil Profiles of Tropical Pasturelands. *Soil Tillage Res* **2024**, *240*, 106100. https://doi.org/10.1016/j.still.2024.106100.

(20) Kim, S.; Kramer, R. W.; Hatcher, P. G. Graphical Method for Analysis of Ultrahigh-Resolution Broadband Mass Spectra of Natural Organic Matter, the Van Krevelen Diagram. *Anal Chem* **2003**, *75* (20), 5336–5344. https://doi.org/10.1021/ac034415p.

(21) Wagner, S.; Brandes, J.; Goranov, A. I.; Drake, T. W.; Spencer, R. G. M.; Stubbins, A. Online Quantification and Compound-Specific Stable Isotopic Analysis of Black Carbon in Environmental Matrices via Liquid Chromatography-Isotope Ratio Mass Spectrometry. *Limnol Oceanogr Methods* **2017**, *15* (12), 995–1006. https://doi.org/10.1002/lom3.10219.

(22) Kappenberg, A.; Bläsing, M.; Lehndorff, E.; Amelung, W. Black Carbon Assessment Using Benzene Polycarboxylic Acids: Limitations for Organic-Rich Matrices. *Org Geochem* **2016**, *94*, 47–51. https://doi.org/10.1016/j.orggeochem.2016.01.009.

(23) Ziolkowski, L. A.; Chamberlin, A. R.; Greaves, J. Quantification of Black Carbon in Marine Systems Using the Benzene Polycarboxylic Acid Method: A Mechanistic and Yield Study. *Limnol Oceanogr Methods* **2011**, No. 4, 140–149.

(24) Stubbins, A.; Spencer, R. G. M.; Mann, P. J.; Holmes, R. M.; McClelland, J. W.; Niggemann, J.; Dittmar, T. Utilizing Colored Dissolved Organic Matter to Derive Dissolved Black Carbon Export by Arctic Rivers. *Front Earth Sci (Lausanne)* **2015**, *3*. https://doi.org/10.3389/feart.2015.00063.

(25) Bostick, K. W.; Zimmerman, A. R.; Wozniak, A. S.; Mitra, S.; Hatcher, P. G. Production and Composition of Pyrogenic Dissolved Organic Matter from a Logical Series of Laboratory-Generated Chars. *Front Earth Sci (Lausanne)* **2018**, *6*, 1–14. https://doi.org/10.3389/feart.2018.00043.

(26) Brodowski, S.; Rodionov, A.; Haumaier, L.; Glaser, B.; Amelung, W. Revised Black Carbon Assessment Using Benzene Polycarboxylic Acids. *Org Geochem* **2005**, *36* (9), 1299–1310. https://doi.org/10.1016/j.orggeochem.2005.03.011.

(27) Dittmar, T. The Molecular Level Determination of Black Carbon in Marine Dissolved Organic Matter. *Org Geochem* **2008**, *39* (4), 396–407. https://doi.org/10.1016/j.orggeochem.2008.01.015.

(28) Schneider, M. P. W.; Hilf, M.; Vogt, U. F.; Schmidt, M. W. I. The Benzene Polycarboxylic Acid (BPCA) Pattern of Wood Pyrolyzed between 200°C and 1000°C. *Org Geochem* **2010**, *41* (10), 1082–1088. https://doi.org/10.1016/j.orggeochem.2010.07.001.

(29) Gustafsson, O.; Haghseta, F.; Chan, C.; Macfarlane John; Gschwend, P. M. Quantification of the Dilute Sedimentary Soot Phase: Implications for PAH Speciation and Bioavailability. *Environ Sci Technol* **1997**, *31*, 203–209. https://doi.org/https://doi.org/10.1021/es960317s.

(30) dos Santos, J. V.; Fregolente, L. G.; Moreira, A. B.; Ferreira, O. P.; Mounier, S.; Viguier, B.; Hajjoul, H.; Bisinoti, M. C. Humic-like Acids from Hydrochars: Study of the Metal Complexation Properties Compared with Humic Acids from Anthropogenic Soils Using PARAFAC and Time-Resolved Fluorescence. *Science of the Total Environment* **2020**, *722*. https://doi.org/10.1016/j.scitotenv.2020.137815.
